# Dimethyl Itaconate Alleviates *Escherichia coli*‐Induced Endometritis Through the Guanosine‐CXCL14 Axis via Increasing the Abundance of *norank_f_Muribaculaceae*

**DOI:** 10.1002/advs.202414792

**Published:** 2025-04-14

**Authors:** Yuhang He, Jiapei Cai, Xufeng Xie, Xinyu Zhang, Linkai Qu, Jiuxi Liu, Yongguo Cao

**Affiliations:** ^1^ State Key Laboratory for Diagnosis and Treatment of Severe Zoonotic Infectious Diseases Key Laboratory for Zoonosis Research of the Ministry of Education Institute of Zoonosis and College of Veterinary Medicine Jilin University Changchun 130062 China; ^2^ Department of Clinical Veterinary Medicine College of Veterinary Medicine Jilin University Changchun 130062 People's Republic of China

**Keywords:** dimethyl itaconate, endometritis, gut microbiota, gut‐uterus axis

## Abstract

Endometritis, a prevalent reproductive system disease with high incidence, leads to reproductive dysfunction in humans and animals, causing huge economic losses. Dimethyl itaconate (DI) has been demonstrated to exert protective effects in multiple inflammatory diseases. Nevertheless, the efficacy of DI in preventing endometritis and the role played by the gut microbiota remain unknown. In this study, it is found that DI ameliorated *Escherichia coli* (*E. coli)* induced endometritis in mice. The protective effect is abolished by antibiotic‐induced depletion of the gut microbiota, and fecal microbiota transplantation (FMT) from DI‐treated mice to recipient mice ameliorated *E. coli*‐induced endometritis. Integrative multiomics reveals that DI promotes the multiplication of *norank_f_Muribaculaceae* in vivo, and supplementation of *Muribaculum intestinale* (DSM 28989), which belongs to the *norank_f_Muribaculaceae* genus, upregulates the level of guanosine in the uterus. Mechanistically, the protective effect of guanosine in endometritis is mediated by activating the expression of *CXCL14* in uterine epithelial cells. Moreover, the antibody‐neutralizing experiment of CXCL14 eliminated this protective effect. In conclusion, this study elucidates the significant role of the gut microbiota and its metabolites in the protection of DI against endometritis, and provides new evidence for the regulation of distal organ by the gut microbiota.

## Graphical abstract

The research findings indicate that dimethyl itaconate (DI) alleviates *Escherichia coli* (*E. coli)* induced endometritis by modulating the gut microbiota and enriching the abundance of *norank_f_Muribaculaceae*, thereby increasing the production of guanosine. The underlying mechanism involves the transportation of the metabolite guanosine from the gut microbiota to the uterine tissue, where it exerts anti‐inflammatory effects by activating CXCL14 in uterine epithelial cells.

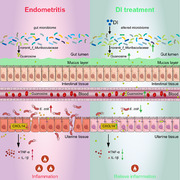

## 1 Introduction

Endometritis, a prevalent reproductive system disease, leads to significant pathological damage to the uterus, resulting in reproductive dysfunction and potential miscarriage in both humans and animals, posing a threat to women's health and causing substantial economic losses in the livestock industry.^[^ ^1^, ^2^ ^]^ Research indicates that approximately 80–90% of uterine infections are attributed to pathogens.^[^ ^3^ ^]^ *Escherichia coli* (*E. coli*) is frequently isolated from the uteri of women and infected dairy cows with endometritis, which is believed to be the primary pathogen responsible for postpartum uterine infections and persistent uterine inflammatory responses.^[^ ^2^, ^4^, ^5^ ^]^ It has been established that the gut microbiota plays a crucial role in infectious disease pathogenesis.^[^ ^6^ ^]^ Furthermore, the gut microbiota has been reported to be closely associated with inflammation and metabolic diseases in both proximal and distal intestinal organs, demonstrating protective effects against pathogenic bacterial infections in murine models of endometritis.^[^ ^7^, ^8^ ^]^ However, the mechanisms of gut microbiota involved in specific microbial and metabolic mediators mediate the development of endometritis are still unclear.

Itaconate, a metabolite generated by immune cell activation via the tricarboxylic acid cycle (TCA cycle), exhibits anti‐inflammatory and immunomodulatory properties.^[^ ^9^ ^]^ Dimethyl itaconate (DI), an itaconate derivative capable of permeating cells, has also demonstrated promising efficacy in both anti‐inflammatory and antibacterial aspects.^[^ ^10^ ^]^ For instance, a mouse model of experimental autoimmune encephalomyelitis (EAE) showed that DI suppressed neuroinflammation.^[^ ^11^ ^]^ DI also possesses the capacity to inhibit the inflammatory response of macrophages in septic mice.^[^ ^12^ ^]^ Mice treated with DI exhibits significantly enhanced resistance to *Staphylococcus aureus* infection and increased survival rates.^[^ ^13^ ^]^

Considering the significant role of gut microbiota and their metabolites in mitigating inflammatory diseases, and the beneficial effects of DI on gut microbiota modulation and amelioration of inflammation, this study aims to investigate the effect of DI in the gut‐uterus axis using an *E. coli*‐induced mouse model of endometritis.^[^ ^8^, ^14^ ^]^ Our research indicates that gavage of DI can significantly increase the abundance of *norank_f_Muribaculaceae* in the gut and increase the generation of the microbial metabolite guanosine, thereby mitigating *E. coli*‐induced endometritis. Therefore, our data provided novel insights into the potential mechanisms through which DI safeguarded the uterus and highlighted the prospective applications of DI in preventing endometritis.

## 2 Results

### 2.1 *E. coli*‐Induced Endometritis was Alleviated by DI in Mice with a Dose‐Dependent Effect

**Figure 1:**
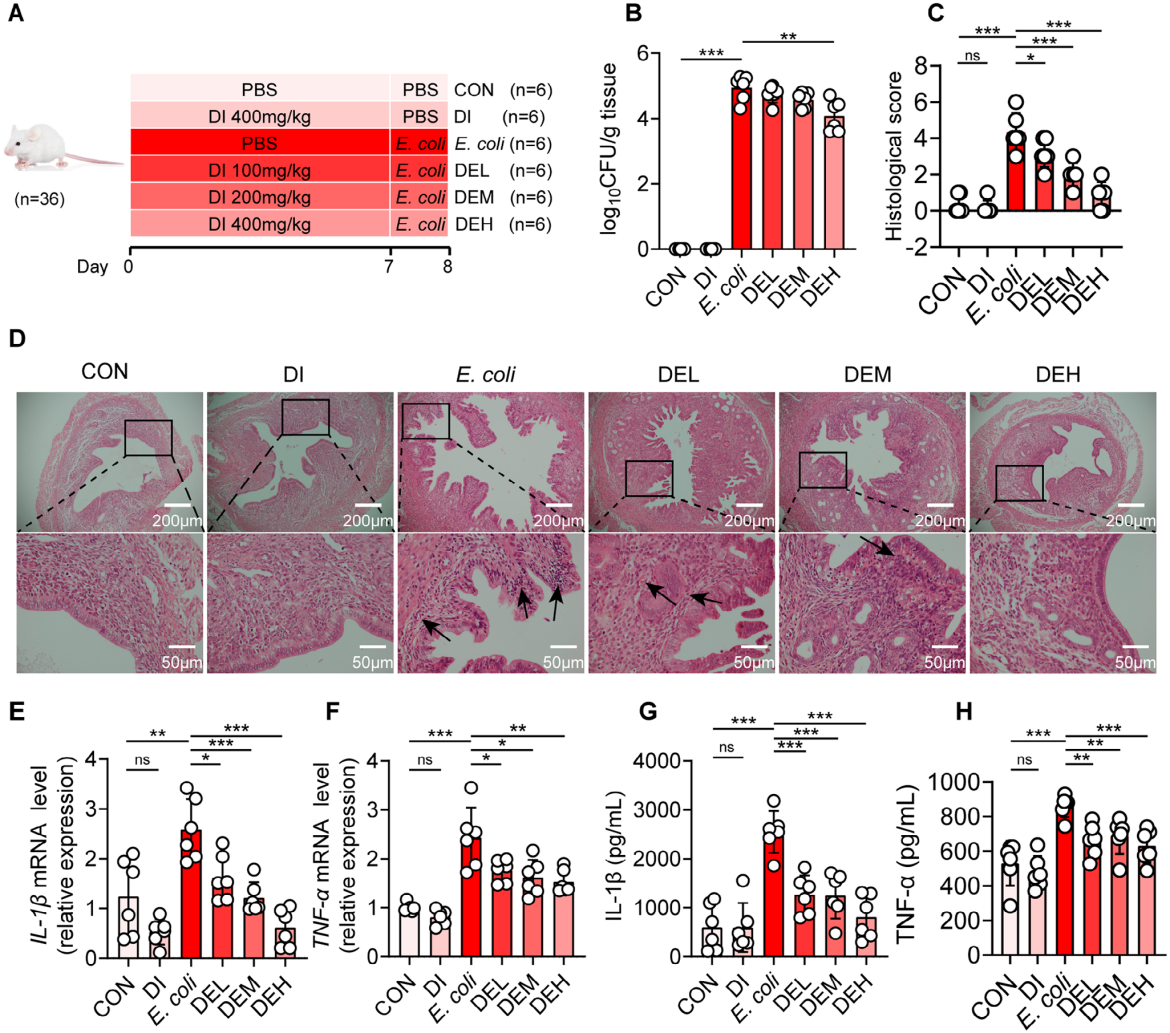
Dimethyl itaconate (DI) dose‐dependently attenuated *E. coli*‐induced endometritis in mice. A–H) The mice were divided into six groups and treat them with different concentrations of DI (0, 100, 200, or 400 mg kg^−1^) for 7 days. Four groups of different concentrations of DI were followed by uterine injection of 10⁸ CFU of *E. coli* to induce endometritis. Six groups were generated: CON, DI (400 mg kg^−1^), *E. coli*, DEL (100 mg kg^−1^ DI+*E. coli*), DEM (200 mg kg^−1^ DI+*E. coli*) and DEH (400 mg kg^−1^ DI+*E. coli*) groups. A) Study design of in vivo mouse experiment. B) The concentration of *E. coli* in the uterus was determined by plate coating (*n* = 6). C) Histological scores in different treatment groups were performed (*n* = 6). D) Representative images of the H&E‐stained uterus sections of indicated groups. The black arrow indicates endometrial injury. E) The mRNA expression of IL‐1β in uterine tissue. F) The mRNA expression of *TNF‐α* in uterine tissue. G) IL‐1β levels in uterine tissue homogenate by ELISA. H) TNF‐α levels in uterine tissue homogenate by ELISA. Data represent means ± SD; ^*^ *p* < 0.05; ^**^ *p* < 0.01; ^***^ *p* < 0.001; by unpaired Student's *t* test. The data shown are representative of three independent experiments.

To determine whether DI can be employed for the prevention of *E. coli*‐induced endometritis, mice were orally administered various concentrations of DI (0, 100, 200, or 400 mg kg^−1^) for 7 days, followed by intrauterine injection of *E. coli* on the final day to induce endometritis (**Figure** **1**A). Compared with the *E. coli* group, the supplementation of DI could result in a dose‐dependent reduction of the bacterial load in the uterus. Specifically, the bacterial count per gram of the uterus in the mice of the *E. coli* group was 114 667 ± 69 244 CFU. After the supplementation of a low dose of DI, the bacterial count per gram of the uterus in the mice decreased to 64 833 ± 29 674 CFU. Under the condition of a medium dose of DI, the bacterial count per gram of the uterus in the mice dropped to 42 333 ± 19 654 CFU. The high dose of DI had the most remarkable effect on reducing the bacterial load in the uterus, with the bacterial count per gram of the uterus in the mice being merely 19 333 ± 18 907 CFU (Figure 1B). Furthermore, compared with the CON group, the uterine tissues in the *E. coli* group manifested severe hyperemia, and neutrophil infiltration was also extremely prominent, forming dense clusters that were easily distinguishable under the microscope. According to the count, its histological score was as high as 4.3 ± 1.03 points. In the DEL group, neutrophil infiltration was partially alleviated, and the normal tissue architecture was partially restored, with a histological score of 3.2 ± 0.75 points. The DEM group further reduced the hyperemia in the uterine tissues, with neutrophil infiltration only occurring in some parts, with a histological score of 2.0 ± 0.63 points. In the DEH group, there was almost no occurrence of uterine hyperemia and neutrophil infiltration, and the histological score was 0.83 ± 0.75 points, approaching a healthy state (Figure 1C,D). Consistently, a reduction in pro‐inflammatory cytokines was observed in mice following oral administration of DI, including IL‐1β (Figure 1E,G) and TNF‐α (Figure 1F,H) as examined by ELISA and Reverse Transcription Quantitative real‐time PCR (RT‐qPCR). These findings suggested that DI alleviated *E. coli*‐induced endometritis in a dose‐dependent manner.

### 2.2 DI Relieved *E. coli*‐Induced Endometritis in a Gut Microbiota‐Depended Manner

We investigated whether the protective role of DI against *E. coli*‐induced endometritis was related with gut microbiota by depleting the commensal microbiota via mixture of antibiotics (ABX) treatment (Figure S1A, Supporting Information). Surprisingly, the ABX+DI+*E. coli* group and the ABX+*E. coli* group mice exhibited indistinguishable abnormal pathological changes, uterine *E. coli* burden, tight junction protein levels, and pro‐inflammatory cytokines levels (Figure S1B–P, Supporting Information). Collectively, our findings suggested that the gut microbiota played a crucial role in mediating the protective effects of DI against endometritis caused by *E. coli*.

**Figure 2:**
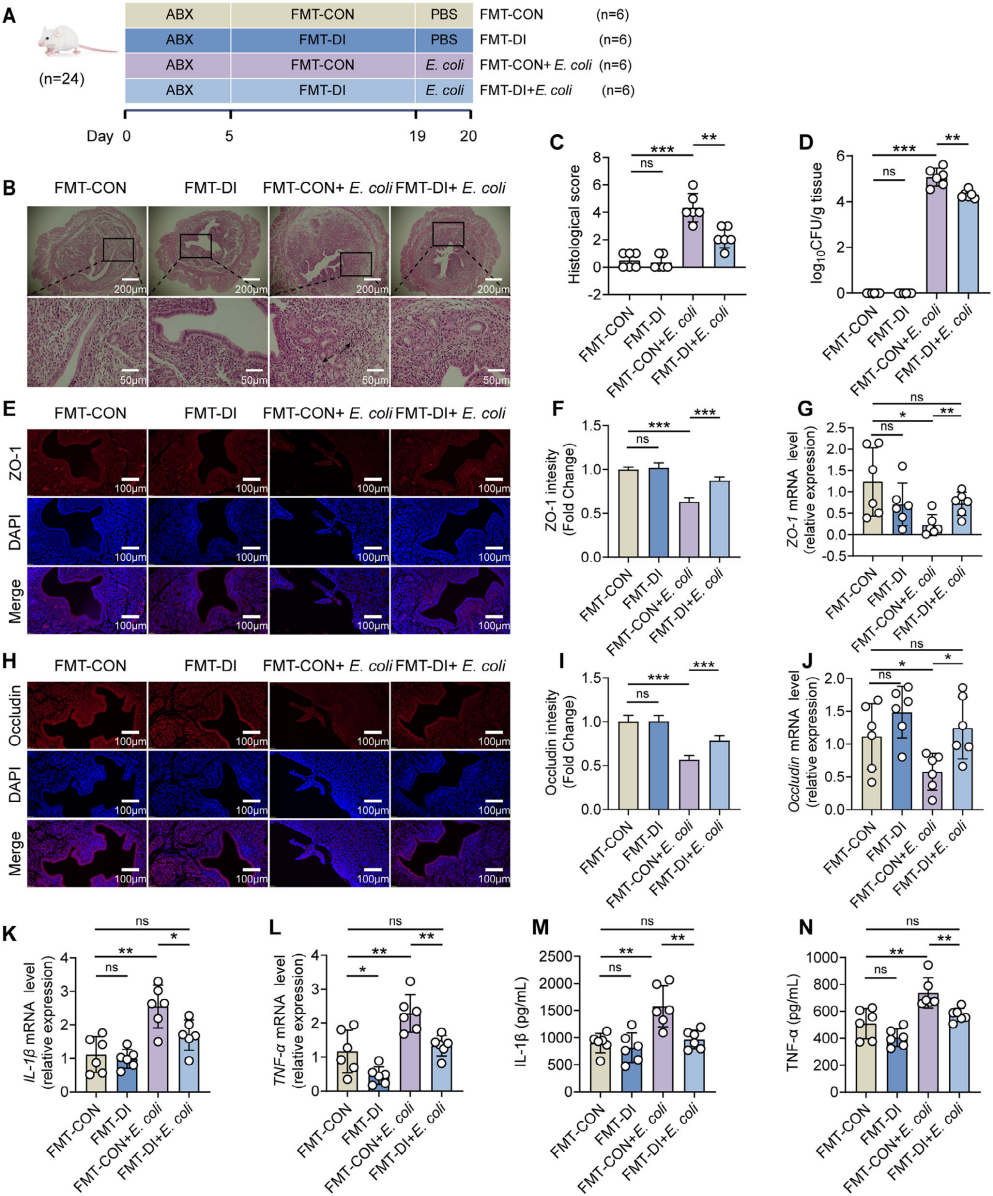
Fecal microbiota transplantation (FMT) from oral administration of dimethyl itaconate (DI) group can alleviate *E. coli*‐induced endometritis in mice. A–N) Mice were administered antibiotics for 5 days to remove gut microbiota, and then colonized with gut microbiota from CON and DI groups for 14 days. Afterwards, the mice were followed by uterine injection of 10⁸ CFU of *E. coli* to induce endometritis (*n* = 6). A) FMT experimental design. B) Representative images of the H&E‐stained uterus sections of indicated groups. The black arrow indicates endometrial injury. C) Histological scores in different treatment groups were performed (*n* = 6). D) The concentration of *E. coli* in the uterus was determined by plate coating. E,F) Uterine sections were immunofluorescent staining with ZO‐1, and the nuclei were visualized by DAPI staining. G) The mRNA expression of *ZO‐1* in uterine tissue. H–I) Uterine sections were immunofluorescent staining with Occludin, and the nuclei were visualized by DAPI staining. J) The mRNA expression of *Occludin* in uterine tissue. K) The mRNA expression of *IL‐1β* in uterine tissue. L) The mRNA expression of *TNF‐α* in uterine tissue. M) IL‐1β levels in uterine tissue homogenate by ELISA. N) TNF‐α levels in uterine tissue homogenate by ELISA. Data represent means ± SD; ^*^ *p* < 0.05; ^**^ *p* < 0.01; ^***^ *p* < 0.001; by unpaired Student's *t* test. The data shown are representative of three independent experiments.

To further explore the causal relationship between DI relieved endometritis and gut microbiota, we executed a fecal microbiota transplantation (FMT) experiment in which mice with depleted gut microbiota were reconstituted with the microbiota from either the CON group or the DI group (**Figure** **2**A). The FMT‐DI+*E. coli* group exhibited significantly reduced abnormal pathological changes, histological score, uterine *E. coli* burden, tight junction protein levels, and pro‐inflammatory cytokines levels, as compared with the FMT‐CON+*E. coli* group (Figure 2B–N, Figure S2A,B, Supporting Information). Specifically, the histological score of mice in the FMT‐CON+*E. coli* group was 4.3 ± 1.03, while that of mice in the FMT‐DI+*E. coli* group remained at a relatively low level of 2.2 ± 0.75 (Figure 2C). Moreover, the bacterial load in the FMT‐CON+*E. coli* group was higher, reaching 162 800 ± 138 738 CFU per gram of uterine tissue. In contrast, the bacterial load in the FMT‐DI+*E. coli* group was significantly decreased to 21 567 ± 9514 CFU per gram of uterine tissue (Figure 2D). Collectively, these results suggested that the gut microbiota was essential in facilitating the protective effects of orally administered DI against endometritis caused by *E. coli* in mice.

### 2.3 DI Altered the Composition of the Gut Microbiota in Mice

**Figure 3:**
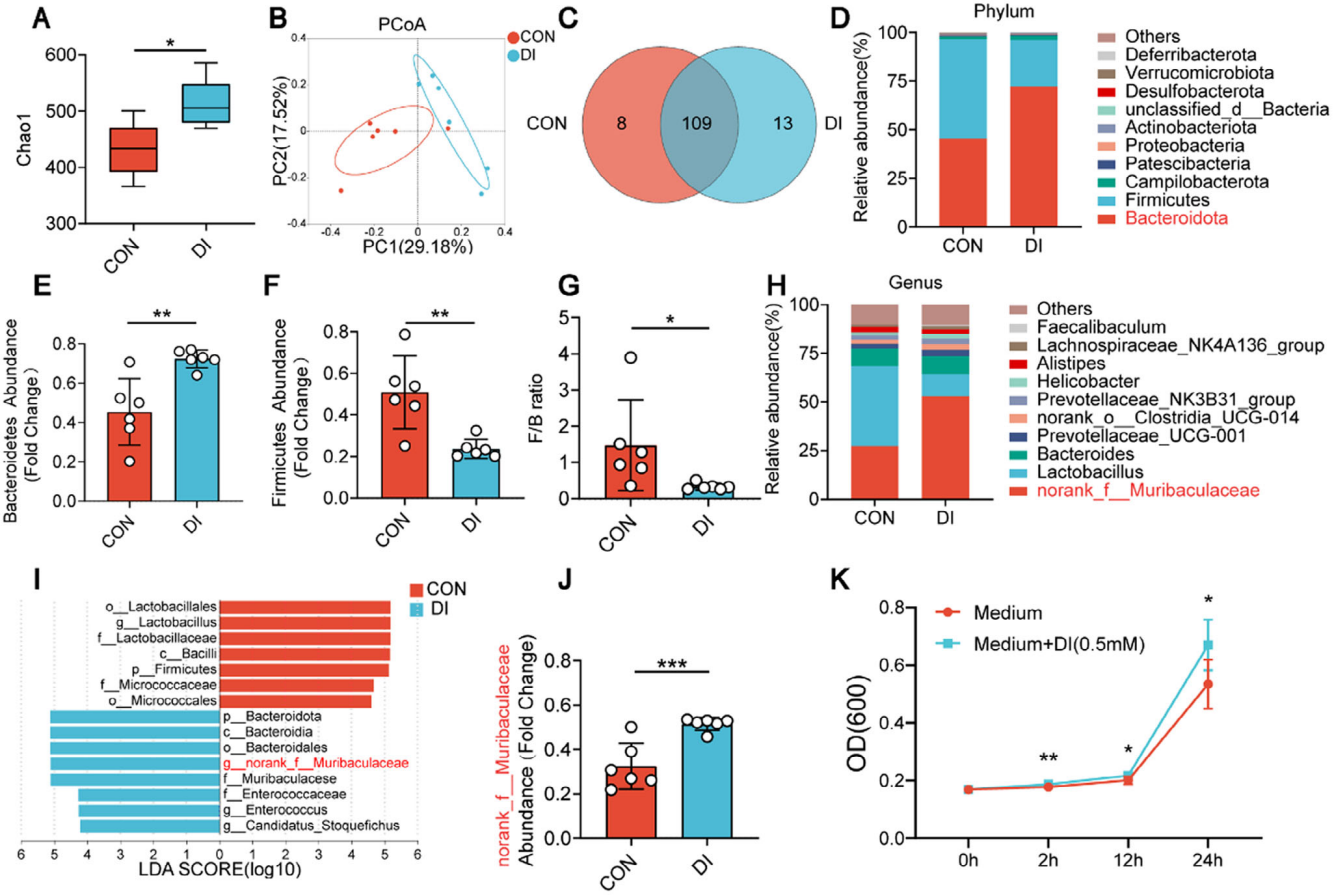
Oral administration of dimethyl itaconate (DI) regulated the composition gut microbiota in mice. A–J) Mice were given DI orally or distilled water for 7 days. Feces from the CON group and DI group mice were collected for 16S rRNA sequencing (*n* = 6). A) The α‐diversity analysis of gut microbes reflected by Chao1 indice. B) Scatter plots of weighted PCoA for the microbial composition showed the differences in gut microbial structure between the CON and DI groups. C) Venn diagram of the amplicon sequence variants (ASVs) in the CON and DI groups. D) Relative abundance of gut microbiota at the phylum level from different treatment groups. E) The abundance of Bacteroidetes between the CON and DI groups. F) The abundance of Firmicutes between the CON and DI groups. G) The ratio of F/B of CON and DI groups. H) Relative abundance of gut microbiota at the genus level from different treatment groups. I) LEfSe showed different bacterial taxa that were enriched in different groups (log10 LDA score >4). J) The abundance of the genera *norank_f_Muribaculaceae* between the CON and DI groups. K) The growth curve of the *M. intestinale* cultured with DI (0.5 × 10^−3^ m). Data represent means ± SD; ^*^ *p* < 0.05; ^**^ *p* < 0.01; ^***^ *p* < 0.001; compared with the CON group.

Our above results showed that the gut microbiota was crucial in preventing *E. coli*‐induced endometritis with oral DI, as demonstrated by ABX and FMT experiments. However, specific modifications in the gut microbiota during this process were unclear. Further investigation into the altered gut microbiota was conducted in the CON group and DI group via 16S rRNA sequencing. Analysis of alpha diversity demonstrated that compared to the CON group, the DI group had higher alpha diversity, indicating that supplementing DI further increased the richness of the gut microbiota (**Figures** **3**A, S3A–C, Supporting Information). partial least squares discriminant analysis (PLS‐DA) and principal coordinate analysis (PCoA) of fecal samples revealed significant variations in gut microbiota communities between the DI group and the CON group (Figure 3B, Figure S3D, Supporting Information). Venn diagrams indicated the presence of eight distinctive amplicon sequence variants (ASVs) within the CON group while there were 13 unique ASVs identified in the DI group (Figure 3C). At the phylum level, the examination of microbiota revealed that Bacteroidetes were more abundant in the DI group, whereas Firmicutes were found to be less prevalent compared to the CON group (Figure 3D–F). Additionally, the DI group demonstrated a notable reduction in the Firmicutes/Bacteroidetes (F/B) ratio (Figure 3G), suggesting that the alteration of gut microbiota through DI might play a role in reducing inflammation.^[^ ^15^ ^]^

Subsequently, we explored the variations at the genus level between the DI group and CON group (Figure 3H). In order to distinguish the differences in bacterial community predominance between these two groups, we performed a comparison and identification using linear discriminant analysis of effect size (LEfSe) (LDA score > 4), exhibiting that DI increases the richness of certain segments of the gut microbiota, with the greatest change observed in *norank_f_Muribaculaceae* (Figure 3I, Figure S3E, Supporting Information). We inspected the specific conditions of the abundance discrepancies of *norank_f_Muribaculaceae* between the two groups within the original data (52.83% versus 27.30%, respectively; *p* < 0.001) (Figure 3J). To investigate the effects of DI on *norank_f_Muribaculaceae* growth, we performed a growth curve analysis supplemented with 0.5 × 10^−3^ m DI.^[^ ^16^ ^]^ The results showed that DI promoted the growth of *M. intestinale*, suggesting that DI provided a favorable environment for the thriving of *M. intestinale* (Figure 3K).

To further validate the potential mechanism of DI on the gut microbiota composition, we performed 16S rRNA gene sequencing to analyze the changes in the gut microbiota composition in mouse feces following a 72‐h anaerobic incubation.^[^ ^17^ ^]^ Consistent with the results of in vivo experiments, *norank_f_Muribaculaceae* showed significant enrichment after the addition of DI during fecal incubation (Figure S4A–G, Supporting Information). Collectively, these data indicated that DI induced alterations in the gut microbiota of mice, and the protective effect of DI against endometritis may be associated with the elevated level of *norank_f_Muribaculaceae*.

### 2.4 *M. intestinale* Alleviated *E. coli*‐Induced Endometritis in Mice

**Figure 4:**
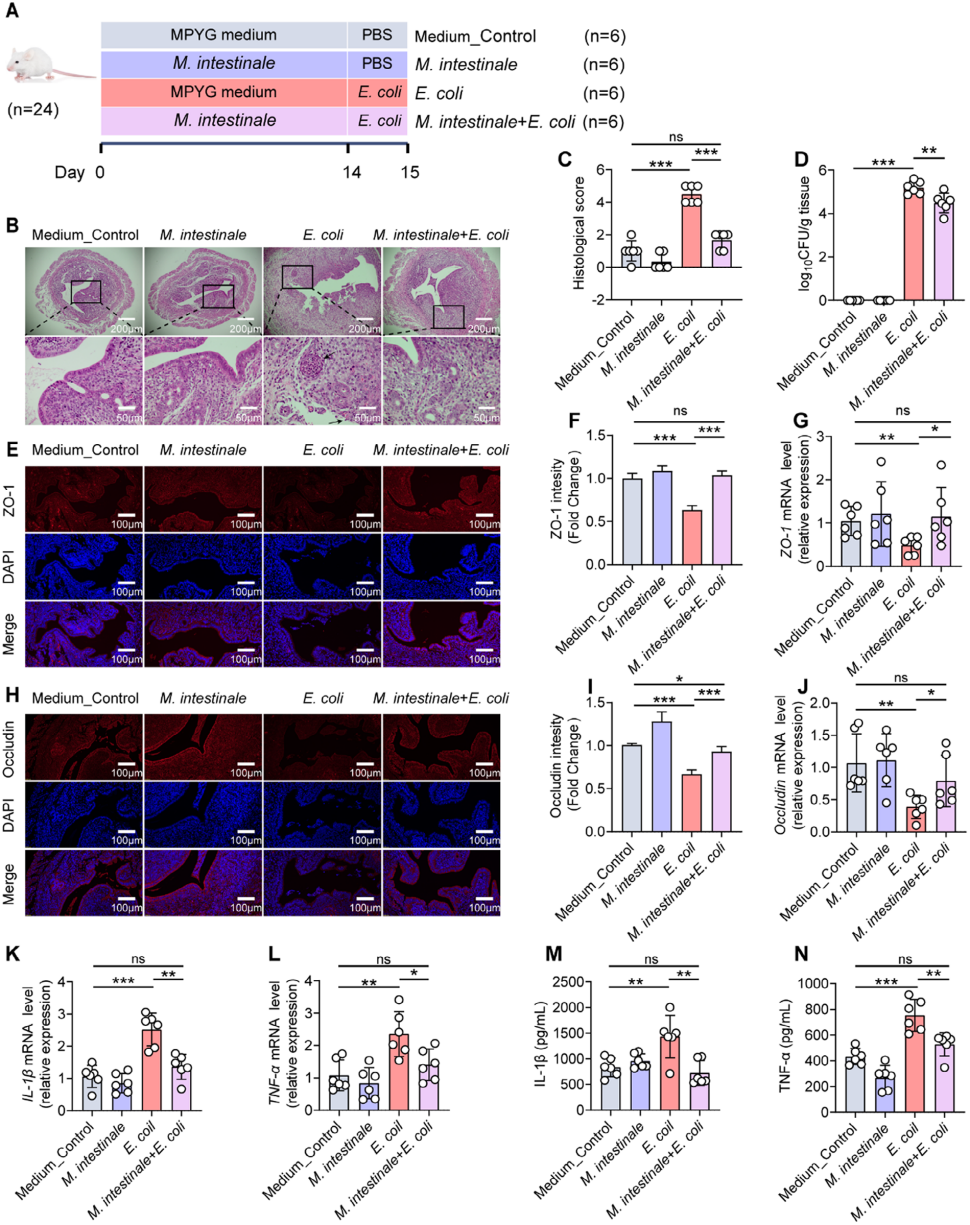
Administration of *M. intestinale* alleviated *E. coli*‐induced endometritis in mice. A–N) Mice were gavaged with *M. intestinale* daily for 2 weeks. Equivalent sterile MPYG medium was used as vehicle control. Afterwards, mice were followed by uterine injection of 10⁸ CFU of *E. coli* to induce endometritis. (A) *M. intestinale* administration experimental design. B) Representative images of the H&E‐stained uterus sections of indicated groups. The black arrow indicates endometrial injury. C) Histological scores in different treatment groups were performed (*n* = 6). D) The concentration of *E. coli* in the uterus was determined by plate coating. E,F) Uterine sections were immunofluorescent staining with ZO‐1, and the nuclei were visualized by DAPI staining. G) The mRNA expression of *ZO‐1* in uterine tissue. H–I) Uterine sections were immunofluorescent staining with Occludin, and the nuclei were visualized by DAPI staining. J) The mRNA expression of *Occludin* in uterine tissue. K) The mRNA expression of *IL‐1β* in uterine tissue. L) The mRNA expression of *TNF‐α* in uterine tissue. M) IL‐1β levels in uterine tissue homogenate by ELISA. N) TNF‐α levels in uterine tissue homogenate by ELISA. Data represent means ± SD; ^*^ *p* < 0.05; ^**^ *p* < 0.01; ^***^ *p* < 0.001; by unpaired Student's *t* test. The data shown are representative of three independent experiments.

To further investigate the role of *norank_f_Muribaculaceae* in endometritis, mice were orally administered with the *M. intestinale* (DSM 28 989), a representative bacterium of the *norank_f_Muribaculaceae* (**Figure** **4**A). Strikingly, mice treated with *M. intestinale* showed a significant decrease in endometritis when compared to those inoculated with the vehicle, including reduced pathological changes, histological score, and uterine *E. coli* burden. Specifically, the histological score decreased from 4.5 ± 0.55 points in the *E. coli* group to 1.7 ± 0.52 points in the *M. intestinale*+*E. coli* group, and the *E. coli* burden decreased from 189 692 ± 128 106 CFU per gram of the uterine tissue in the *E. coli* group to 45 867 ± 44 588 CFU per gram of the uterine tissue in the *M. intestinale*+*E. coli* group (Figure 4B–D). Unlike mice that were administered vehicle treatment, those treated with *M. intestinale* demonstrated a reduction in inflammation, characterized by an increase in tight junction proteins, zonula occludens‐1 (ZO‐1) and Occludin, and a decrease in pro‐inflammatory cytokines (such as TNF‐α and IL‐1β). (Figure 4E–N, Figure S5A,B, Supporting Information). These findings indicated that administration of *M. intestinale* alleviated endometritis in mice.

### 2.5 DI Altered Gut Microbiota‐Derived Purine Metabolism and Promoted the Enrichment of Guanosine

**Figure 5:**
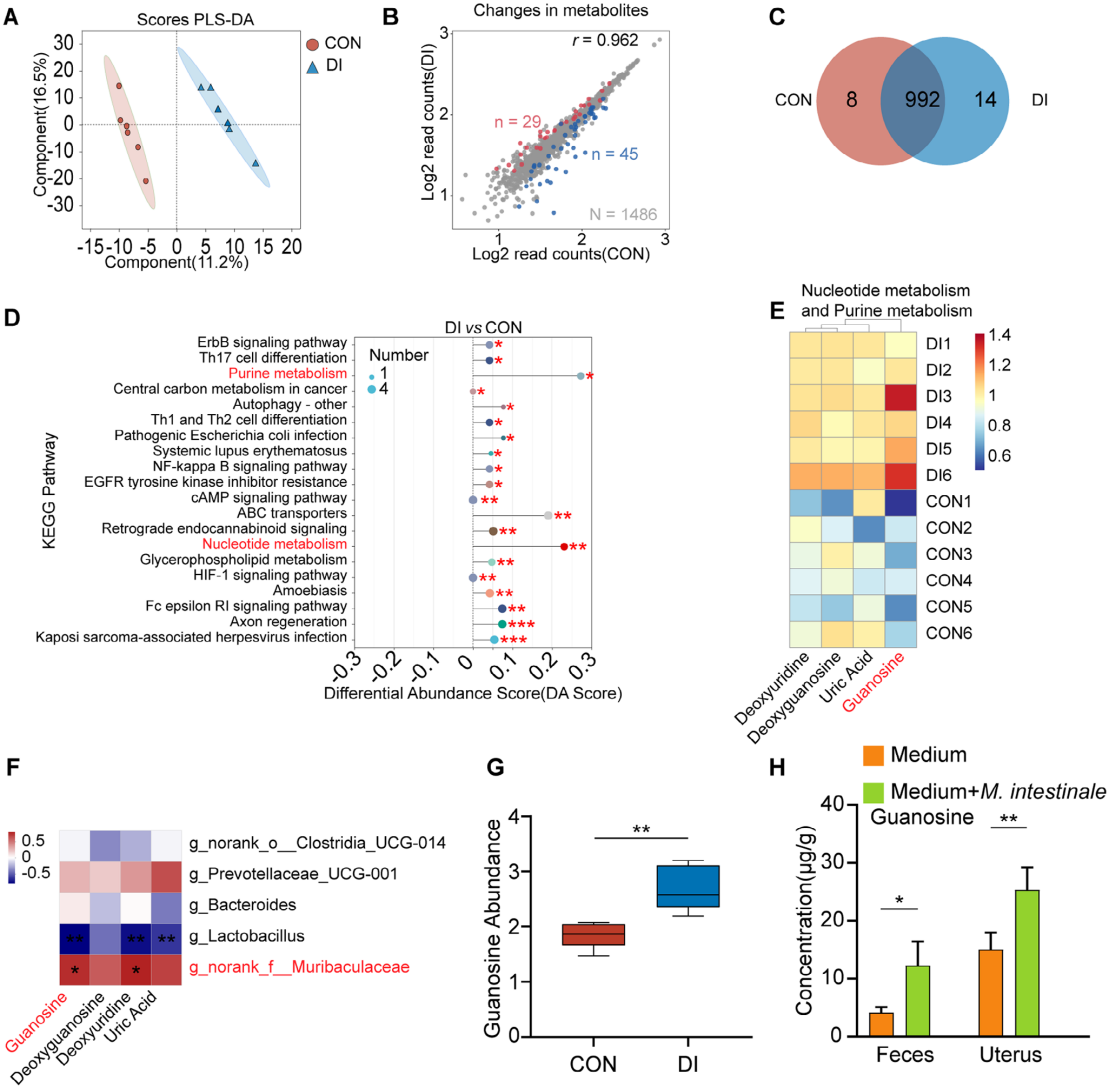
Dimethyl itaconate (DI) altered the metabolic profiling and enriched microbiota‐derived purine metabolites–guanosine. A–G) Mice were orally administered distilled water or DI for 7 days. Feces from the CON group and DI group mice were collected for untargeted metabolomics (*n* = 6). A) Partial least‐squares discriminant analysis (PLS‐DA) was performed on the metabonomics profiles in fecal samples from the DI and CON groups. B) Volcano plot of differentially expressed genes. C) Venn diagrams. D) KEGG pathway difference abundance score chart. E) Heat map of significantly altered metabolites in feces. F) Correlation analysis between fecal metabolites exhibiting significant changes and differential gut microbiota. G) Distribution box plot of metabolite guanosine. H) The abundance of guanosine in the feces and uterus of mice from *M. intestinale* administration experiment (3–4 biological replicates for each group). The red color denotes a positive correlation, while blue color denotes a negative correlation. The intensity of the color is proportional to the strength of Spearman correlation. Data represent means ± SD; ^*^ *p* < 0.05; ^**^ *p* < 0.01; ^***^ *p* < 0.001; by unpaired Student's *t* test.

To identify the pivotal metabolites and metabolic pathways involved in alterations of oral DI in mice, untargeted metabolomics was employed for fecal analysis of the DI group and CON group. The PLS‐DA models demonstrated a significant distinction in cluster separation between the two groups (**Figure** **5**A). Compared with the CON group, the feces of the DI group exhibited 29 significantly increased metabolites and 45 metabolites were notably decreased (Figure 5B). Additionally, Venn diagrams revealed 8 unique metabolites in the CON group while the DI group exhibited 14 unique metabolites (Figure 5C). It was noteworthy that the KEGG pathway difference abundance score chart highlighted the significant impact of DI on purine metabolism and nucleotide metabolism pathways (Figure 5D). Notably, guanosine and its downstream product deoxyguanosine were commonly enriched metabolites in these two pathways. Further analysis was conducted on the four differential metabolites common to the two significantly enriched metabolic pathways between the DI group and the CON group. The results indicated that the levels of all four differential metabolites were elevated in the samples of the DI group compared to those of the CON group. Among them, guanosine was the most significantly differentially enriched metabolite (Figure 5E, Figure S6A, Supporting Information), and it showed a significant positive correlation with the differential gut bacteria of the *norank_f_Muribaculaceae* (Figure 5F). Consistently, distribution box plot of metabolites showed that DI treatment significantly elevated guanosine abundance compared to that of the CON group (Figure 5G). LC‐MS/MS revealed that guanosine was substantially increased in the feces and uterus after intestinal colonization with gavage *M. intestinale* (Figures 5H). Our findings from untargeted metabolomic analysis and LC‐MS/MS demonstrated that the supplementation of DI or *M. intestinale* elevated the level of guanosine that might be an important mediator in the gut‐uterus axis during endometritis.

### 2.6 DI Alleviated *E. coli*‐Induced Endometritis by Elevating the Levels of Guanosine

**Figure 6:**
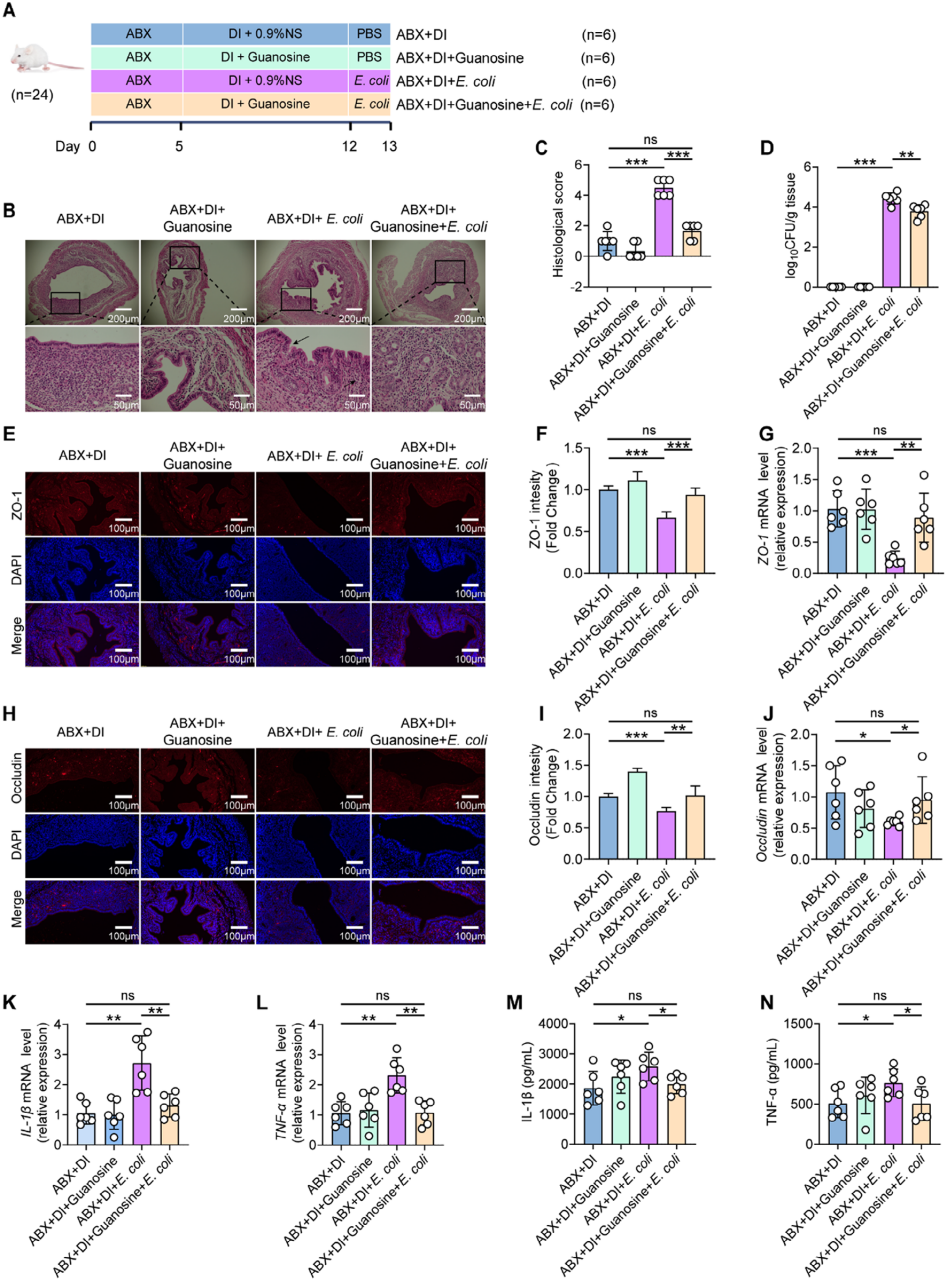
Dimethyl itaconate (DI), rather than directly exerting anti‐inflammatory effects, mediates protective effects against *E. coli*‐induced endometritis through the metabolite guanosine produced by the gut microbiota. A–N) Mice were pretreated with ABX for 5 days to deplete the gut microbiota. Subsequently, DI was administered orally. Meanwhile, guanosine dissolved in 0.9% normal saline (0.9% NS) at a dose of 8 mg kg^−1^ was given by intraperitoneal injection for 7 days, followed by uterine injection with 10^8^ CFU of *E. coli* to induce endometritis (*n* = 6). A) Guanosine supplementation experimental design. B) Representative images of the H&E‐stained uterus sections of indicated groups. The black arrow indicates endometrial injury. C) Histological scores in different treatment groups were performed (*n* = 6). D) The concentration of *E. coli* in the uterus was determined by plate coating. E,F) Uterine sections were immunofluorescent staining with ZO‐1, and the nuclei were visualized by DAPI staining. G) The mRNA expression of *ZO‐1* in uterine tissue. H–I) Uterine sections were immunofluorescent staining with Occludin, and the nuclei were visualized by DAPI staining. J) The mRNA expression of *Occludin* in uterine tissue. K) The mRNA expression of *IL‐1β* in uterine tissue. L) The mRNA expression of *TNF‐α* in uterine tissue. M) IL‐1β levels in uterine tissue homogenate by ELISA. N) TNF‐α levels in uterine tissue homogenate by ELISA. Data represent means ± SD; ^*^ *p* < 0.05; ^**^ *p* < 0.01; ^***^ *p* < 0.001; by unpaired Student's *t* test. The data shown are representative of three independent experiments.

Considering the significant increase in microbial metabolite guanosine following oral administration of DI or *M. intestinale*, we postulated that the preventive effect of DI or *M. intestinale* on *E. coli*‐induced endometritis is mediated by the enrichment of gut microbiota‐derived guanosine. To investigate this hypothesis, we administered antibiotic mixtures to deplete the gut microbiota and subsequently orally administered DI with or without guanosine. This allowed us to assess whether the abolished preventive effect of DI on endometritis after the depletion of gut microbiota could be reversed by the supplementation of guanosine (**Figure** **6**A). Histological analysis revealed that mice in the ABX+DI+*E. coli* group exhibited more severe damage, manifested as more severe hyperemia and increased infiltration of immune cells, resulting in a histological score of 4.5 ± 0.55; whereas those in the ABX+DI+Guanosine+*E. coli* group showed significant alleviation of uterine tissue damage, with the histological score significantly dropping to 1.7 ± 0.52 (Figure 6B,C). The bacterial load in the ABX+DI+*E. coli* group was as high as 31 300 ± 18 526 CFU per gram of tissue. In contrast, the ABX+DI+guanosine+*E. coli* group exhibited a lower bacterial burden, with only 7467 ± 4168 CFU per gram of tissue (Figure 6D). Consistently, the treatment of guanosine also reversed the decrease in concentration and expression levels of tight junction protein, include ZO‐1 (Figure 6E–G), and Occludin (Figure 6H–J, Figure S7A,B, Supporting Information), compared with those in the ABX+DI+*E. coli* group. The ABX+DI+Guanosine+*E. coli* group reduced endometritis inflammatory markers, including IL‐1β (Figure 6K,M) and TNF‐α (Figure 6L,N) concentrations and expression level, compared with the ABX+DI+*E. coli* group. These findings suggested that the preventive effect of DI on *E. coli*‐induced endometritis was not directly attributed to its impact on the uterus, but rather to the enrichment of guanosine in the gut microbiota.

### 2.7 DI Modulated the Uterine Gene Expression Profile and Activated the Cytokine‐Cytokine Receptor Interaction Pathway

**Figure 7:**
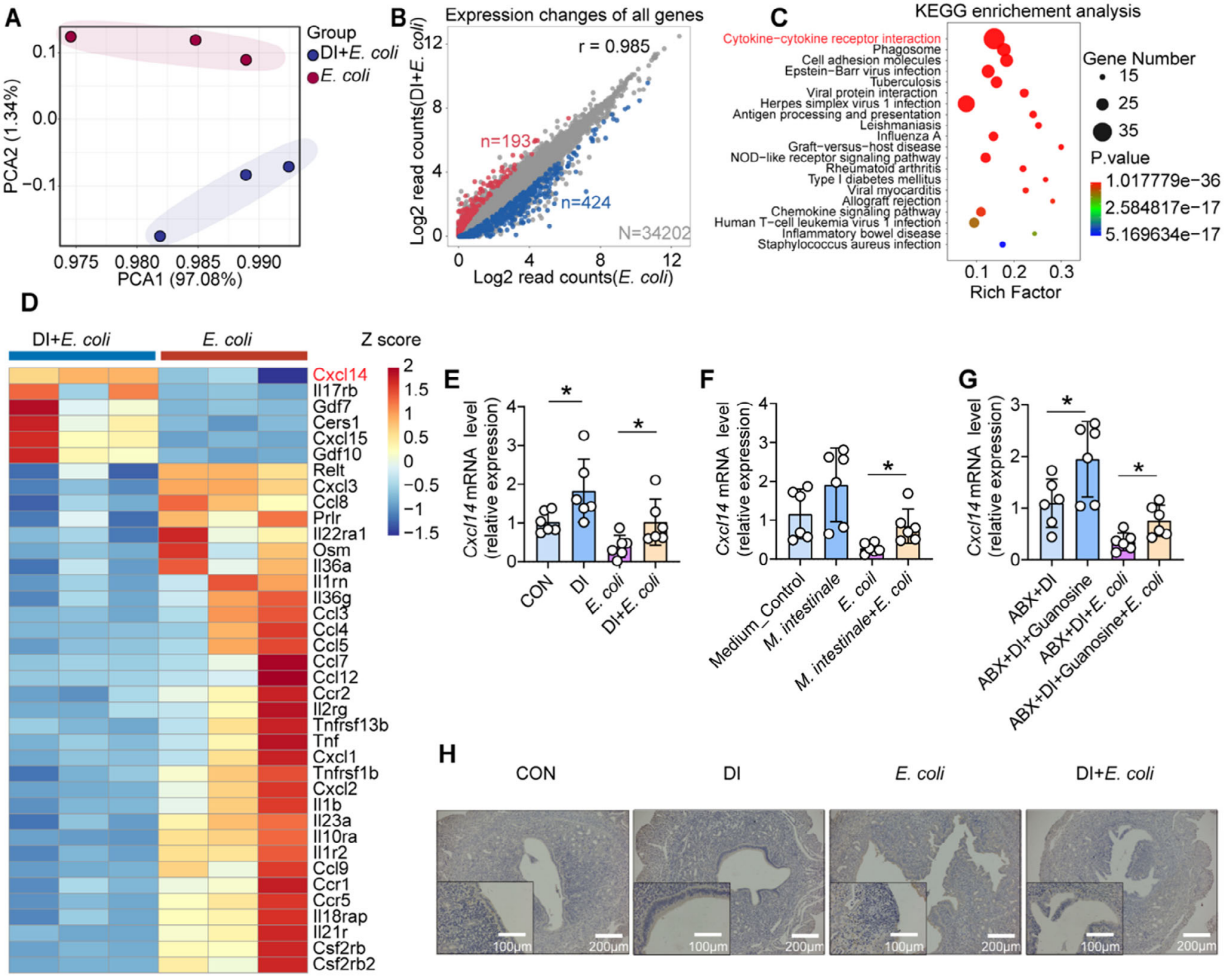
Dimethyl itaconate (DI) alters gene expression in the mouse uterus. A–H) Mice were orally administered distilled water or DI for 7 days, followed by induction of endometritis using *E. coli* (*n* = 3). A) Principal component analysis (PCA) of the transcriptome of mouse uterine tissue. B) Dot plot of differentially expressed genes in the uterus after oral administration of DI. C) Enrichment analysis of the most significantly altered pathways in the KEGG pathway. D) Heatmap analysis of the enriched differential genes in the cytokine‐cytokine receptor interaction pathway. E–G) Relative expression changes of CXCL14 in the previous experiments by RT‐qPCR. H) Representative images of immunohistochemistry staining with CXCL14. Data are mean ± SD. ^*^ *p* < 0.05, ^**^ *p* < 0.01, and ^***^ *p* < 0.001. Statistical analysis was performed using Student's *t* test.

To further elucidate the potential mechanism by which oral DI ameliorates *E. coli*‐induced endometritis in mice, RNA sequencing was conducted on the entire uterine tissue. A significant difference in the transcriptome was observed between the DI+*E. coli* group and the *E. coli* group (**Figure** **7**A). Dot plot depicted the transcriptional changes in all genes, revealing that the DI+*E. coli* group versus the *E. coli* group exhibited 424 downregulated genes and 193 upregulated genes (Figure 7B). KEGG pathway analysis revealed that within the DI+*E. coli* group, the cytokine–cytokine receptor interaction emerged as the most significantly enriched functional pathway among the signaling pathways analysis (Figure 7C). Interestingly, among the 38 significantly altered genes in the cytokine–cytokine receptor interaction pathway, CXCL14 exhibited the most pronounced upregulation in the DI+*E. coli* group (Figure 7D).

Using RT‐qPCR and Western blot (WB), we confirmed the expression of the key gene *CXCL14* in the cytokine–cytokine receptor interaction pathway, and observed that the expression of CXCL14 was significantly upregulated in mice after oral administration of DI, regardless of whether *E. coli* was used to induce endometritis (Figure 7E, Figure S8A,B, Supporting Information). When the gut microbiota was depleted using ABX, the upregulation of the gene was no longer observed (Figure S9A, Supporting Information). With the supplementation of DI donor mouse FMT‐DI, CXCL14 was also upregulated (Figure S9B, Supporting Information). In addition, gavage with *M. intestinale* or guanosine also led to a significant upregulation of CXCL14 expression (Figure 7F–G, Figure S8C–F, Supporting Information). The immunohistochemical staining results showed that both DI and guanosine increased the expression of CXCL14 in the uterus, mainly expressed by uterine epithelial cells (Figure 7H, Figure S9C, Supporting Information). These results demonstrated that CXCL14 was a pivotal gene modulated by DI. In conclusion, gavage of DI led to changes in genes related to uterine tissue, and the activation of CXCL14 might be associated with the enhancement of endometritis treatment by DI.

### 2.8 Guanosine Ameliorated *E. coli*‐Induced Murine Endometritis via CXCL14

**Figure 8:**
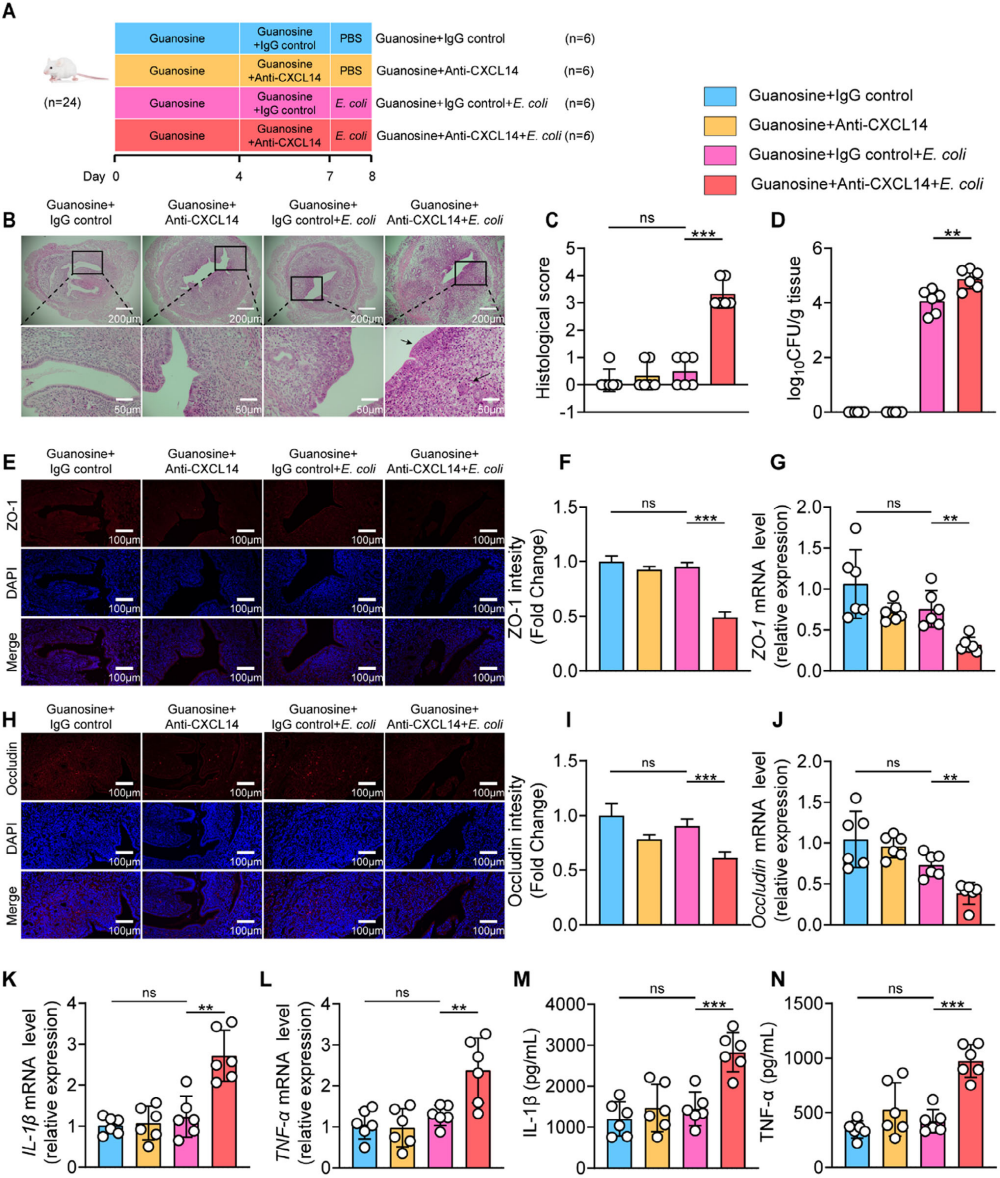
The protective effect of guanosine against *E. coli*‐induced endometritis disappears when systemic neutralization of CXCL14 is performed. A–N) Mice received intraperitoneal injections of guanosine for seven consecutive days, followed by intraperitoneal injection of Anti‐CXCL14 for the last 3 days to neutralize endogenous CXCL14. Subsequently, mice were followed by uterine injection of 10⁸ CFU of *E. coli* to induce endometritis. A) Experimental design for CXCL14 inhibition. B) Representative images of the H&E‐stained uterus sections of indicated groups. The black arrow indicates endometrial injury. C) Histological scores in different treatment groups were performed (*n* = 6). D) The concentration of *E. coli* in the uterus was determined by plate coating. E,F) Uterine sections were immunofluorescent staining with ZO‐1, and the nuclei were visualized by DAPI staining. G) The mRNA expression of *ZO‐1* in uterine tissue. H–I) Uterine sections were immunofluorescent staining with Occludin, and the nuclei were visualized by DAPI staining. J) The mRNA expression of *Occludin* in uterine tissue. K) The mRNA expression of *IL‐1β* in uterine tissue. L) The mRNA expression of *TNF‐α* in uterine tissue. M) IL‐1β levels in uterine tissue homogenate by ELISA. N) TNF‐α levels in uterine tissue homogenate by ELISA. Data represent means ± SD; ^*^ *p* < 0.05; ^**^ *p* < 0.01; ^***^ *p* < 0.001; by unpaired Student's *t* test. The data shown are representative of three independent experiments.

The RNA‐seq results highlighted an essential role of CXCL14 in the protective effect of guanosine against *E. coli*‐induced endometritis. To investigate whether CXCL14 mediates the protective effect of guanosine in ameliorating *E. coli*‐induced mouse endometritis, we employed an anti‐CXCL14 monoclonal antibody which is capable of neutralizing CXCL14 in vivo to assess the impact of CXCL14 on the protective effect of guanosine in improving *E. coli*‐induced endometritis (**Figure** **8**A). As expected, mice treated with guanosine were safeguarded against *E. coli*‐induced endometritis, including fewer pathological changes, reduced histological scores, lower uterine *E. coli* burden, increased tight junction protein levels, and decreased pro‐inflammatory cytokine concentrations (Figure 8B–N, Figure S10A,B, Supporting Information). The histological score of the Guanosine+IgG control+*E. coli* group was only 0.5 ± 0.55 points, and the bacterial burden was merely 16 267 ± 11 980 CFU per gram of tissue. However, the neutralization of CXCL14 eliminated the protective effect of guanosine on endometritis caused by *E. coli*, which was manifested by an increase in the histological score to 3.3 ± 0.52 and an elevation of the bacterial load to 97 033 ± 66 152 CFU per gram of the uterus (Figure 8B–N, Figure S10A,B, Supporting Information). Overall, our data suggested that the protective role of guanosine against endometritis is at least partially mediated by CXCL14.

## 3 Discussion

A growing body of evidence suggests that imbalances in gut microbiota can lead to disruptions in microbial metabolites, which are associated with the onset of various diseases, including those affecting distant organs.^[^ ^18^ ^]^ Research on the gut‐brain axis, gut‐lung axis, and gut‐liver axis, elucidates how the gut microbiota signals to peripheral organs and influence physiological processes and disease pathogenesis.^[^ ^19^, ^20^, ^21^, ^22^, ^23^, ^24^, ^25^ ^]^ While initial studies have suggested that the gut microbiota plays a role in preventing endometritis, the exact mechanism by which the gut microbiota influences endometritis through microbial metabolites remains unclear.^[^ ^26^ ^]^ In the current investigation, we observed that the supplementation of itaconate derivative–DI was capable of alleviating *E. coli*‐induced endometritis and reconfiguring the gut microbiota. The underlying mechanism involved enriching the gut microbiota with beneficial bacteria *norank_f_Muribaculaceae* to enhance production of microbial metabolite guanosine, thereby activating anti‐inflammatory factor CXCL14. Our research findings further substantiated the existence of the gut‐uterus axis and underscored the pivotal role played by the gut microbiota and its derivatives in endometritis pathogenesis, offering novel theoretical underpinning for modulating the gut microbiota to exert remote organ control.

Itaconate has the ability to inhibit the production of pro‐inflammatory cytokines, showing anti‐inflammatory properties both in vitro and in vivo, while also exhibiting strong antibacterial effects.^[^ ^9^, ^27^, ^28^ ^]^ Its derivative DI exhibits membrane permeability and possesses various functionalities such as anti‐inflammatory effects and immune regulation—rendering it an exceedingly promising preventive and therapeutic agent.^[^ ^29^, ^30^ ^]^ For instance, under hyperlipidemic conditions, DI alleviates inflammation in C2C12 myocytes through the AMPK/FGF21/PPARδ signaling pathway.^[^ ^31^ ^]^ Young and colleagues discovered that pre‐treating murine bone marrow‐derived macrophages with DI reduces the production of pro‐inflammatory cytokines triggered by lipopolysaccharide (LPS) and also prevents the activation of the NLRP3 inflammasome caused by nigericin.^[^ ^32^ ^]^ However, the majority of studies have utilized intraperitoneal injection, which presents challenges for widespread application in clinical and livestock production.^[^ ^33^, ^34^ ^]^ Conversely, oral administration, while more convenient, has received comparatively less attention. In this study, we found that administering DI orally can mitigate induced endometritis by modulating the gut microbiota and promoting guanosine production.

The intestines contain a vast and varied microbial population that is essential for preserving gut balance and is associated with several conditions, including obesity, liver disorders, brain issues, cardiovascular diseases, and inflammatory bowel diseases.^[^ ^35^, ^36^, ^37^ ^]^ Increasing evidences suggest that the anti‐inflammatory effects of drugs are associated with the modulation of the gut microbiota.^[^ ^38^, ^39^ ^]^ Previous studies reported that following the addition of DI, there was a restructuring of the mouse gut microbiota, marked by greater microbial diversity, an increase in Bacteroidetes phylum bacteria, and a decrease in Firmicutes phylum bacteria.^[^ ^14^ ^]^ In line with previous studies, our 16S rRNA gene sequencing revealed that DI significantly improved the richness and diversity of the gut microbiota in mice, while concomitantly reducing the F/B ratio. An elevation in Bacteroidetes can ameliorate inflammation by stimulating regulatory T cells to secrete IL‐10, which is an anti‐inflammatory cytokine.^[^ ^40^ ^]^ Additionally, Bacteroidetes has been found to exhibit negative correlations with plasma inflammation markers TNF‐α, C‐reactive protein (CRP), and lipopolysaccharide binding protein (LBP) underscoring its significance in inflammation.^[^ ^41^ ^]^ Following oral administration of DI, we observed a significant increase in the prevalence of *norank_f_Muribaculaceae* within the Bacteroidetes phylum. The anaerobic fermentation experiment of mouse feces yielded consistent results, demonstrating an increase in the abundance of the *norank_f_Muribaculaceae* upon addition of DI. Prior research indicated a close correlation between *norank_f_Muribaculaceae* and the formation of the mucus layer, as well as its involvement in maintaining barrier function within the colon.^[^ ^42^ ^]^ A decrease in the levels of *norank_f_Muribaculaceae* correlates with increased colitis severity in mouse models.^[^ ^43^ ^]^

The substantial body of evidence demonstrated the capacity of gut microbiota‐derived metabolites to modulate the physiology and pathology of distant organs, such as the pancreas,^[^ ^44^ ^]^ brain,^[^ ^45^, ^46^ ^]^ and liver.^[^ ^47^ ^]^ Thus, we explored the relationship between DI‐mediated alleviation of endometritis and the metabolic pathways of the gut microbiota. Subsequent KEGG pathway analysis revealed differential expression levels in multiple metabolic pathways between the DI and CON groups, especially in purine metabolism and nucleotide metabolism. Inflammatory states are associated with the extracellular release of nucleotides, especially ATP.^[^ ^48^ ^]^ Adenosine originates from intracellular purine metabolism and nucleotide metabolism, and a reduction in its signaling can result in increased production of inflammatory cytokines.^[^ ^49^ ^]^ Allopurinol in purine metabolism can inhibit xanthine oxidase to improve mixed granulocytic airway inflammation induced by toluene diisocyanate.^[^ ^50^ ^]^ Notably, purine metabolism and nucleotide metabolism are enriched with common metabolites including guanosine and its downstream product deoxyguanosine. Guanosine‐based multidrug‐loaded codelivery system has demonstrated the ability to suppress typical inflammatory cytokines such as TNF‐α and IL‐6, and ameliorate inflammatory bowel disease through synergistic interactions among its components.^[^ ^51^ ^]^ Studies previously demonstrated that guanosine demonstrates favorable effects in rats with DNBS‐induced colitis by modulating colonic inflammation and suppressing NF‐κB‐mediated signaling.^[^ ^52^ ^]^ Consistent with these studies, untargeted metabolomics results demonstrated a significant elevation in guanosine levels following DI supplementation. Interestingly, we confirmed that supplementation with guanosine effectively alleviated the endometritis phenotype induced by *E. coli*. The dosage of guanosine supplementation, 8 mg kg^−1^, was determined by referring to previous literature.^[^ ^52^, ^53^ ^]^ This finding suggests that the beneficial effects of DI on endometritis at least partially depend on the production of guanosine.

RNA sequencing, a high‐throughput sequencing technology, generates extensive transcriptome data; after preprocessing, comparison, quantification and analysis, it identifies differentially expressed genes and conducts functional enrichment via bioinformatics to unveil the panorama of gene expression and changes in a tissue.^[^ ^54^, ^55^ ^]^ Next, we applied RNA‐seq to investigate the potential mechanisms by which DI improves uterine inflammation. KEGG analysis revealed a significant enrichment of the cytokine‐cytokine receptor interaction pathway following DI supplementation. Notably, heatmap analysis highlighted CXCL14 as the gene with the most significant differences within this pathway, serving as a multifunctional anti‐inflammatory target.^[^ ^56^, ^57^ ^]^ Previous studies have shown that CXCL14 is a key chemokine that plays a crucial role in regulating infection and immune responses, facilitating the recruitment of macrophages and their polarization towards an M2‐like phenotype to exert anti‐inflammatory effects.^[^ ^58^ ^]^ In line with these findings, our data confirmed the anti‐inflammatory role of CXCL14; oral administration of DI and guanosine significantly enhanced the secretion of CXCL14 in endometrial epithelial cells and alleviated endometritis, while systemic neutralization of CXCL14 eliminated the protective effect against endometritis.

Overall, our results demonstrate that administration of DI significantly ameliorated *E. coli*‐induced endometritis and reshaped the gut microbiota in mice. The participation of the gut microbiota in the protective effect of DI on endometritis was further confirmed through depletion of the symbiotic microbiota, FMT, and 16S ribosomal RNA (rRNA) sequencing. Moreover, DI was found to significantly promote the abundance of the *norank_f_Muribaculaceae* (Its previous name was the S24‐7 family belonging to the Bacteroidetes phylum) in the gut.^[^ ^19^, ^26^ ^]^ We identified a purine metabolite, guanosine, derived from the gut microbiota, which mediated the beneficial effects of DI in reducing uterine inflammation. Furthermore, RNA sequencing (RNA‐seq) and immunohistochemical analysis demonstrated that the beneficial effects of DI on endometritis was attributed to the upregulation of CXCL14 expression in uterine epithelial cells. These discoveries underline that the regulation of distal organs mediated by the intestinal microbiota and metabolites represents a promising therapeutic approach and offers an alternative management for uterine disorders.

## 4 Conclusions

In conclusion, our research attempts to provide new insights into how the gut microbiota regulates distant organs such as the uterus. We found that DI alleviated *E. coli*‐induced endometritis by modulating the gut microbiota and enriching the abundance of *norank_f_Muribaculaceae*, leading to increased guanosine production. The underlying mechanism involved the transportation of metabolite guanosine from the gut microbiota to the uterine tissue, where they exerted anti‐inflammatory effects through the activation of CXCL14 in uterine epithelial cells. Our findings validated the presence of the gut‐uterus axis and emphasized the essential contribution of the gut microbiota and its metabolites to endometritis pathogenesis, offering a novel theoretical framework for modulating diseases in distant organs through the gut microbiota.

## 5 Experimental Section

### Animals

Female BALB/c mice (8 weeks) were obtained from Liaoning Changsheng Biotechnology Co., Ltd., located in Benxi, China. All mice were maintained at room temperature (20–24 °C) and housed on a 12 h light/dark cycle. The mice were used for experiments after a week of adaptive feeding with ample water and breeding fodder. All animal studies received approval from the Institutional Animal Care and Use Committee (IACUC) of Jilin University (KT202302207).

### Materials

*Escherichia coli* CVCC1418 was sourced from the China Veterinary Culture Collection Center (CVCC) and inoculated into Luria‐Bertani (LB) broth medium (Cat# HB0128) which was purchased from HOPEBIO (Qingdao, China) and cultured at 37 °C in an oscillating incubator at 180 rpm for 12 h. *Muribaculum intestinale* (DSM 28 989) was acquired from the Leibnitz Institute DSMZ‐German Collection of Microorganisms and Cell Cultures and cultivated in MPYG Medium (Cat# HB8931) which was purchased from HOPEBIO (Qingdao, China) supplemented with 3% FBS (TransGen Biotech, Beijing, China), and grown anaerobically (≈5% H_2_, 85% N_2_, 10% CO_2_) at 37 °C for 3 days.

The primary reagents utilized in this research, DI (Cat# 617 527) and guanosine (Cat# 118 003), were acquired from Sigma‐Aldrich (St. Louis, MO, USA). Mouse polyclonal antibodies CXCL14 (Cat# GTX108431) were purchased from GeneTex (SC, USA). Mouse CXCL14/BRAK antibody (Cat# MAB730) were purchased from R&D System (Minneapolis, MN, USA). Enzyme‐linked immunosorbent assay (ELISA) kits for tumor necrosis factor α (TNF‐α) (Cat# 430 901) and interleukin 1β (IL‐1β) (Cat# 432 601) were sourced from Biolegend (San Diego, CA, USA). Specific antibodies including Cy3‐conjugated Goat Anti‐Rabbit IgG (Cat# GB21303) was purchased from Servicebio (Wuhan, China). HRP‐conjugated Goat Anti‐Rabbit IgG(H+L) (Cat# SA00001‐2), β‐actin polyclonal antibody (Cat# 20536‐1‐AP), ZO‐1 (Cat# 21773‐1‐AP), and Occludin (Cat# 27260‐1‐AP) were acquired from proteintech (Wuhan, China).

### Establishment of the Endometritis Model

A mouse model of endometritis induced by *E. coli* infection was created following the methods outlined previously.^[^ ^59^, ^60^ ^]^ In brief, after being anesthetized with ethyl carbamate, the mouse uterus was injected with 10^8^ CFU of *E. coli* at a volume of 60 µL using a 100 µL syringe equipped with a 30‐gauge blunt needle (30 µL on both sides). Twenty‐four hours posttreatment with either *E. coli* or PBS, the cervixes and fallopian tubes of the mice were carefully transected. Subsequently, the entire uterine bodies, along with the bilateral uterine horns, were harvested. These samples were then promptly stored in a −80 °C freezer to maintain their integrity for subsequent analyses of inflammation‐related parameters.

### Antibiotic Cocktail (ABX) Experiment

From the same batch of female BALB/c SPF mice received an oral administration of a four‐antibiotic combination (neomycin at 200 mg kg^−1^, metronidazole at 200 mg kg^−1^, ampicillin at 200 mg kg^−1^, and vancomycin at 100 mg kg^−1^) for five consecutive days to eliminate the gut microbiota.^[^ ^61^, ^62^ ^]^ All antibiotics utilized in the study were obtained from Sigma‐Aldrich (neomycin: Cat# 1405‐10‐3, metronidazole: Cat# 443‐48‐1, ampicillin Cat# 69‐52‐3, vancomycin: Cat# 1404‐93‐9; St. Louis, MO, USA). Subsequently, the mice were subjected to oral gavage of DI for seven consecutive days, and on the final day, they were treated with *E. coli* to induce an endometritis model.

### CXCL14 Inhibition Experiment

The specific procedure of the CXCL14 inhibition experiment is as previously described but with some slight modifications.^[^ ^63^, ^64^ ^]^ The procedure has been adjusted as follows: In the experimental group, mice received intraperitoneal injections of guanosine for seven consecutive days. Additionally, in the last 3 days, they were administered 5 mg kg^−1^ of the anti‐CXCL14 antibody via intraperitoneal injection to neutralize endogenous CXCL14. In contrast, the control group of mice were treated with an isotype‐matched IgG control. On the final day of the experiment, all mice in the control group were treated with *E. coli* to induce the endometritis model.

### Fecal Microbiota Transplantation (FMT) Experiment

From the same batch of female BALB/c SPF mice received an oral administration of a four‐antibiotic combination (neomycin at 200 mg kg^−1^, metronidazole at 200 mg kg^−1^, ampicillin at 200 mg kg^−1^, and vancomycin at 100 mg kg^−1^) for five consecutive days to eliminate the gut microbiota. Feces were obtained from donor mice (CON and DI groups), reconstituted in PBS to a concentration of 0.125 g mL^−1^, and subsequently administered to the recipient mice via oral gavage at a volume of 0.2 mL once daily for a duration of 14 days. Then, the mice were treated with *E. coli* to induce endometritis model.

### Uterine Bacterial Load Measurement

To evaluate the *E. coli* load in the uterus of different treatment groups, 0.05 g of uterine tissue was weighed in a relatively aseptic environment. Specifically, the uterus was first unfolded and cut longitudinally into two halves. Subsequently, one uterine horn and half of the uterine body were weighed, with appropriate adjustments made until a total weight of 0.05 g was reached, ensuring an equal proportion of uterine horn and body tissues. Next, 500 µL of sterile PBS was added to the tissue sample, which was then homogenized at low temperature to yield a 10% tissue homogenate. Subsequently, 50 µL of this homogenate was evenly spread on LB agar plates. After 16‐h incubation, the colonies on the plates were counted to determine the bacterial load. The remaining homogenate was centrifuged in a low‐temperature environment, and the supernatant was collected for subsequent ELISA assays.

### Histological Scores of Uterine Sections

Uterine tissue samples from different treatment groups intended for histological analysis were preserved in 4% paraformaldehyde for a minimum of 48 h. Then embed the fixed sample in paraffin and cut it into 5‐µm sections (6 per mouse). Following dewaxing and hydration with xylene and alcohol, the sections were stained with hematoxylin and eosin (H&E) and examined using an optical microscope (Olympus, Tokyo, Japan). Uterine tissue samples from different treatment groups intended for histological analysis were preserved in 4% paraformaldehyde for a minimum of 48 h. Then embed the fixed sample in paraffin and cut it into 5‐µm sections (6 per mouse). Following dewaxing and hydration with xylene and alcohol, the sections were stained with H&E and examined using an optical microscope (Olympus). Histological score of the uterine tissue was conducted following a previously reported protocol.^[^ ^65^ ^]^ Key histopathological parameters, namely hyperemia and neutrophil infiltration, were assessed. Hyperemia was graded on a scale from 0 to 3, where 0 denoted normal conditions and 3 indicated severe hyperemia, with intermediate grades of mild and moderate in between. Neutrophil infiltration was graded on a scale from 0 to 5, higher grades representing increasing degrees of infiltration.

### Extraction of Total RNA and RT‐qPCR

Total RNA was isolated from the uterine tissue of mice utilizing the RNAiso Plus reagent (Cat# 9109, Takara Bio, Inc., Otsu‐Shiga, Japan) and quantified by RT‐qPCR. In short, a tissue sample weighing 30 mg was extracted with 1 mL of Trizol and processed with chloroform, isopropanol, and 75% ethanol in RNase‐free conditions. Following reverse transcription with TransStart Tip Green qPCR SuperMix (Beijing, China), cDNA was amplified using specific primers and FastStart Universal SYBR Green Master Mix (ROX) (Roche, Switzerland, Basel) in a Step One Plus instrument (Applied Biosystems, Foster City, CA, USA). GAPDH was utilized as a reference control, and all data were adjusted relative to the control group. The primers employed in this research are listed in Appendix 1 Table S1 (Supporting Information).

### Western Blotting

The total proteins were extracted from uterine tissue samples using tissue protein extraction reagents (Thermo Fisher Scientific, USA). Target proteins were separated by 8%, 10%, or 15% SDS‐PAGE according to their molecular weights, and subsequently transferred onto a 0.45‐µm PVDF membrane. After blocking with 5% skimmed milk at room temperature for 3 h, target proteins at appropriate final concentrations were detected using specific antibodies, including Occludin, CXCL14, and β‐actin, following the manufacturer's instructions. The PVDF membrane was then incubated with goat anti‐rabbit IgG. After washing with TBST, proteins were visualized using an ECL Plus Western Blotting Detection System (Tanon, Chna).

### Inflammatory Cytokine Analysis

The concentrations of the proinflammatory cytokines TNF‐α and IL‐1β in uterine tissues were measured using an ELISA kit (TNF‐α: Cat# 430 901, IL‐1β: Cat# 432 601, Biolegend, CA, USA), adhering to the instructions provided by the manufacturer. All standard curves, which were fitted using a four‐parameter logistic model provided by the manufacturer, exhibited *R* ^2^ values at or near 1. The concentrations of cytokines were derived from these standard curves.

### In Vitro Fermentation System

Feces of untreated mice were collected. The samples were promptly gathered into a tightly closed tube and placed in an anaerobic workstation. The feces from all mice were equally divided into six portions (1 g), and suspended in 50 mL of fermentation medium (MPYG medium supplemented with 3% FBS). Three of them were added with 0.5 × 10^−3^ m DI, while the other three were supplemented with an equal volume of PBS.^[^ ^16^ ^]^ The obtained suspension was homogenized in a blender and filtered through a 100‐mesh filter to remove larger particles. Subsequently, the centrifuge tubes containing the fecal suspension were placed in an anaerobic culture bag (Cat# HBYY007) and subjected to anaerobic culture in dark at 37 °C for 72 h. Then, the cultures were collected for examination.

### Gavage *M. intestinale* to Mice

To explore the protective effect of *M. intestinale* on endometritis in mice, a bacterial gavage experiment was conducted. The dose of the *M. intestinale* was adjusted to 2 × 10^8^ CFU per 200 µL, and mice were gavaged with 200 µL daily for 14 consecutive days. Subsequently, mice were followed by uterine injection of 10⁸ CFU of *E. coli* to induce endometritis.

### Immunofluorescence Measurement

Following fixation in 4% paraformaldehyde and embedding in paraffin, the uterine tissue was sliced into sections of 6 µm thickness using a cryostat. Subsequently, the samples underwent two rounds of dewaxing with xylene (Servicebio, Wuhan, China) for 20 min each time, soak twice in 100% and 95% ethanol for 10 min each time, and finally soak twice in distilled water for 3 min each time. Following a 30‐min steaming in sodium citrate buffer (pH = 6.0, Servicebio, Cat# G1201‐1L) for antigen retrieval. Washing twice with PBS buffer solution, the sections were incubated with 5% BSA (Sigma‐Aldrich, St. Louis, MO, USA) for 1 h to block nonspecific binding and subsequently treated with the specific primary antibody at 4 °C overnight. Leave at room temperature for 20 min to restore temperature and washing with PBS, the appropriate fluorescent secondary antibody was applied in the dark for 1 h. Ultimately, the sections were washed twice with PBS buffer solution and then incubated with DAPI for 15 min. Fluorescent proteins in the uterus were observed, and images were obtained with a fluorescence microscope (Olympus) for later analysis using software.

### Immunohistochemistry

The paraffin‐embedded uterine sections were immersed in xylene and a series of ethanol concentrations for dewaxing and hydration. Following antigen retrieval, an immunohistochemical hypersensitivity kit (KIT‐9710, Maxin Biotechnologies, Fuzhou, China) was utilized according to the manufacturer's guidelines. Subsequently, the samples were incubated overnight at 4 °C with mouse polyclonal antibodies against CXCL14 (Cat# GTX108431). The following day, the samples underwent three washes in PBS, after which the kit was employed for the subsequent steps. The chromogenic agent for the enzyme substrate (DAB‐031, Maxin Biotechnologies) was applied to develop the color of the sections. Lastly, hematoxylin was utilized to stain the nuclei, and neutral resin was employed for mounting.

### Extraction of Genomic DNA from Fecal Samples and Sequencing of 16S Ribosomal RNA (rRNA)

Genomic DNA from total microbial communities was extracted from frozen fecal samples using the TIANamp Stool DNA Kit (TIANGEN, Cat. No. DP328, Beijing, China) following the manufacturer's guidelines. The DNA's quality and concentration were evaluated through 1.0% agarose gel electrophoresis and a NanoDrop2000 spectrophotometer (Thermo Scientific), with the samples being preserved at −80 °C for future use. The hypervariable V3‐V4 region of the bacterial 16S rRNA gene was amplified using the primer pair 338F (5′‐ACTCCTACGGGAGGCAGCAG‐3′) and 806R (5′‐GGACTACHVGGGTWTCTAAT‐3′) on a T100 Thermal Cycler PCR machine from BIO‐RAD, USA. The resulting PCR product was extracted from a 2% agarose gel and purified according to the manufacturer's guidelines with the PCR Clean‐Up Kit provided by YuHua in Shanghai, China. Subsequently, quantification was performed using Qubit 4.0 from Thermo Fisher Scientific. The purified amplicons were combined in equal molar ratios and subsequently sequenced using a paired‐end method on the Illumina PE300/PE250 platform (Illumina, San Diego, USA), following the standard protocols established by Majorbio Bio‐Pharm Technology Co. Ltd. (Shanghai, China). Operational taxonomic units (OTUs) were clustered using a 97% similarity threshold through UPARSE version 7.1, during which chimeric sequences were detected and eliminated. The classification of each representative sequence from the OTUs was conducted with RDP Classifier version 2.2, referencing the 16S rRNA database and applying a confidence level of 0.7. The similarities in microbial communities among different samples were assessed through Principal Coordinates Analysis (PCoA) using Bray‐Curtis dissimilarity, implemented via the Vegan v2.5‐3 package. To pinpoint bacterial taxa that exhibited significant enrichment across various treatment groups, LEfSe analysis was conducted with criteria of LDA score greater than 2 and *p*‐value less than 0.05. Additionally, the Shannon and Chao1 indices were calculated to evaluate alpha diversity. A Wilcoxon rank‐sum test was conducted to determine the bacterial taxa that exhibited significant differences between the two groups, with a false discovery rate set at below 0.05.

### Untargeted Metabolomics

A metabolomic analysis was conducted using LC‐MS/MS by Majorbio in Shanghai, China. In summary, 50 mg of the fecal sample was combined with 400 µL of an extraction solvent composed of methanol and water in a 4:1 ratio. Additionally, this solution included an internal standard at a concentration of 0.02 mg mL^−1^ (L‐2‐chlorophenylalanine). Following the processes of grinding, ultrasound treatment, and centrifugation, the supernatant obtained was transferred into an injection vial for analysis via LC‐MS/MS. This analysis was conducted using a Thermo UHPLC‐Q Exactive HF‐X system equipped with an ACQUITY HSS T3 column (100 mm × 2.1 mm i.d., 1.8 µm; Waters, USA) at Majorbio Bio‐Pharm Technology Co. Ltd., situated in Shanghai, China. Data was collected using the Data Dependent Acquisition (DDA) method. Detection was conducted across a mass range of 70 to 1050 m/z. Subsequently, the R package “ropls” (Version 1.6.2) was employed for principal component analysis (PCA) and orthogonal partial least squares discriminant analysis (OPLS‐DA), which included a 7‐cycle interactive validation process to assess the model's stability. Metabolites with a VIP score exceeding 1 and a *p*‐value below 0.05 were considered significantly different, as determined by the Variable Importance in Projection (VIP) from the OPLS‐DA model, along with *p*‐values calculated using Student's *t* test. The metabolites that showed differences between the two groups were then associated with their biochemical pathways through metabolic enrichment and pathway analysis using the KEGG database. Metabolites can be categorized based on their associated pathways or distinct functions. An enrichment analysis was conducted to determine whether a specific group of metabolites is linked to a particular functional node.

### RNA‐seq Analysis of Gene Expression

The mRNA sequencing was performed at LC‐Bio Technology Co., Ltd. located in Hangzhou, China. To summarize, total RNA was isolated from uterine tissue samples utilizing TRIzol reagent (Invitrogen, CA, USA). To analyze the transcriptome data, RNA libraries were generated and sequenced using 2 × 150 bp paired‐end sequencing (PE150) on an Illumina Novaseq 6000 platform. The DESeq2 package from Bioconductor was utilized to conduct the differential expression analysis of gene centers.

### Statistical Analysis

Statistical analyses were performed using GraphPad Prism 8 (San Diego, CA, USA), presenting results as mean ± SD or in boxplot format. An unpaired Student's *t* test (parametric) was employed to assess significant differences between the two groups. A *p*‐value less than 0.05 is considered significant in all statistical analyses. Additional detailed evaluations can be found in the figure legends.

## Conflict of Interest

The authors declare no conflict of interest.

## Supporting information



Supporting Information

Supporting Information

Supporting Information

## Data Availability

The data that support the findings of this study are available from the corresponding author upon reasonable request.
