# Supplementary material for: Dimethyl Itaconate Alleviates Escherichia coli‐Induced Endometritis Through the Guanosine‐CXCL14 Axis via Increasing the Abundance of norank_f_Muribaculaceae
Source: Adv Sci (Weinh). 2025 Apr 14;12(21):2414792. doi: 10.1002/advs.202414792 (PMC12140315; doi:10.1002/advs.202414792)
Supplement: Supplementary file 1 — Supporting Information [file ADVS-12-2414792-s003.docx]

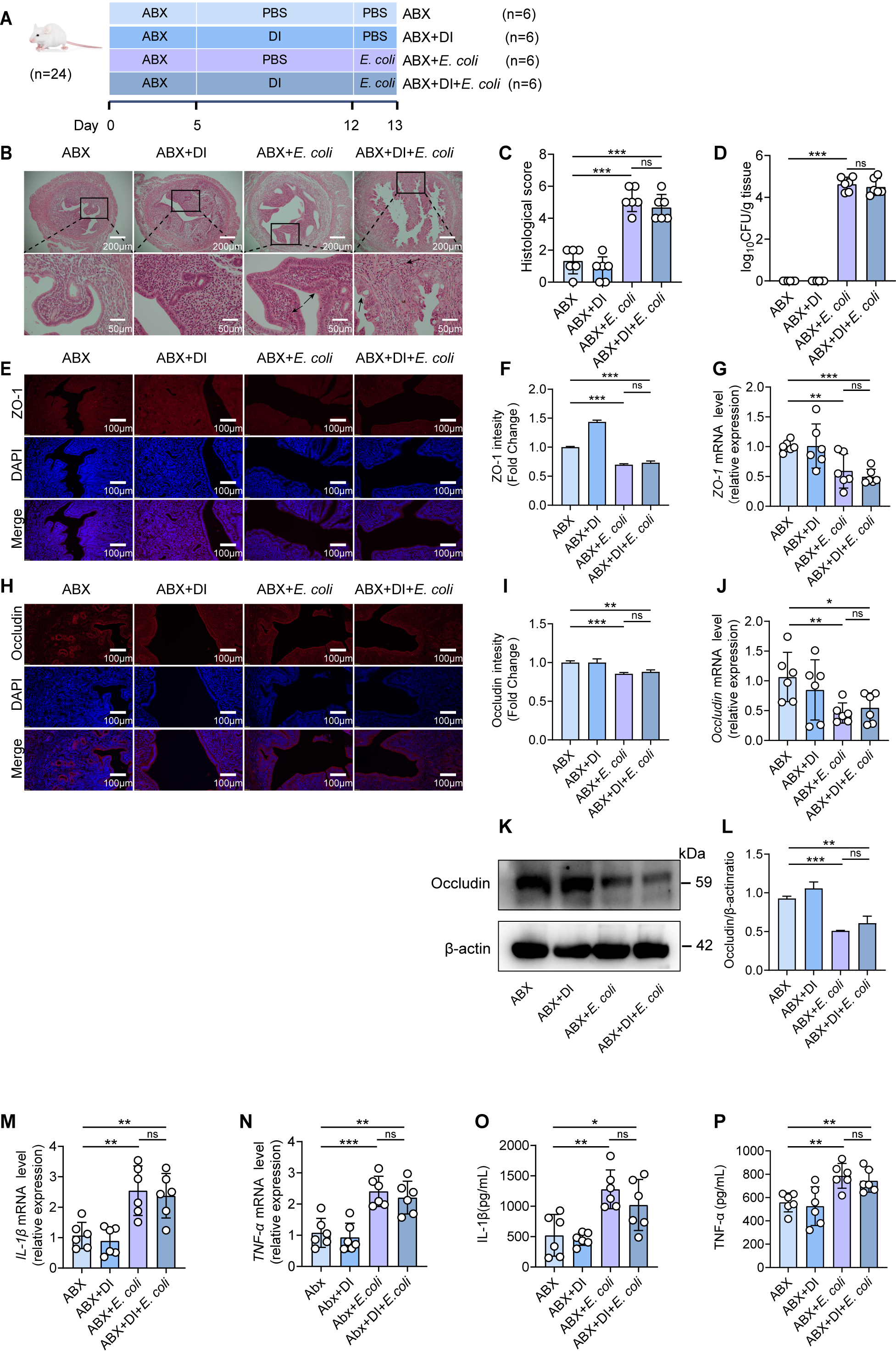


**Supplemental Figure 1.** Depletion of the gut microbiota impairs oral administration of DI mediated protective effects on *E. coli*-induced endometritis in mice. **(A-P)** Pretreated mice with ABX for 5 days to deplete the gut microbiota prior to oral administration of DI for 7 days, followed by *E. coli*-induced endometritis (n = 6). **(A)** ABX experimental design. **(B)** Representative images of the H&E-stained uterus sections of indicated groups. The black arrow indicates endometrial injury. **(C)** Histological scores in different treatment groups were performed(n=6). **(D)** The concentration of *E. coli* in the uterus was determined by plate coating. **(E-F)** Uterine sections were immunofluorescent staining with ZO-1, and the nuclei were visualized by DAPI staining. **(G)** The mRNA expression of *ZO-1* in uterine tissue. **(H-I)** Uterine sections were immunofluorescent staining with Occludin, and the nuclei were visualized by DAPI staining. **(J)** The mRNA expression of *Occludin* in uterine tissue. **(K)** Representative Western blot images of Occludin in the uteri of mice and **(L)** analysis of the relative intensities (n = 3). **(M)** The mRNA expression of *IL-1β* in uterine tissue. **(N)** The mRNA expression of *TNF-α* in uterine tissue. **(O)** IL-1β levels in uterine tissue homogenate by ELISA. **(P)** TNF-α levels in uterine tissue homogenate by ELISA. Data represent means ± SD; **P* < 0.05; ***P* < 0.01; ****P* < 0.001; by unpaired Student’s *t* test. The data shown are representative of three independent experiments


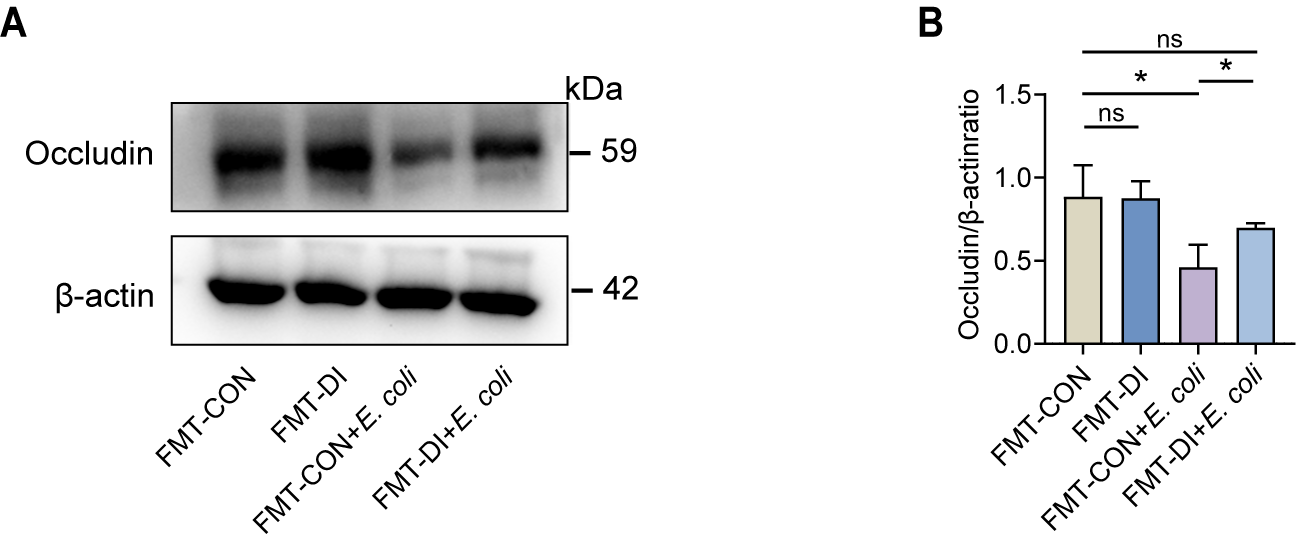


**Supplemental Figure 2.** Changes in the levels of the tight junction protein Occludin in the fecal microbiota transplantation (FMT) experiment. **(A)** Representative Western blot images of Occludin in the uteri of mice and **(B)** analysis of the relative intensities (n = 3). Data represent means ± SD; **P* < 0.05; ***P* < 0.01; ****P* < 0.001; by unpaired Student’s *t* test.


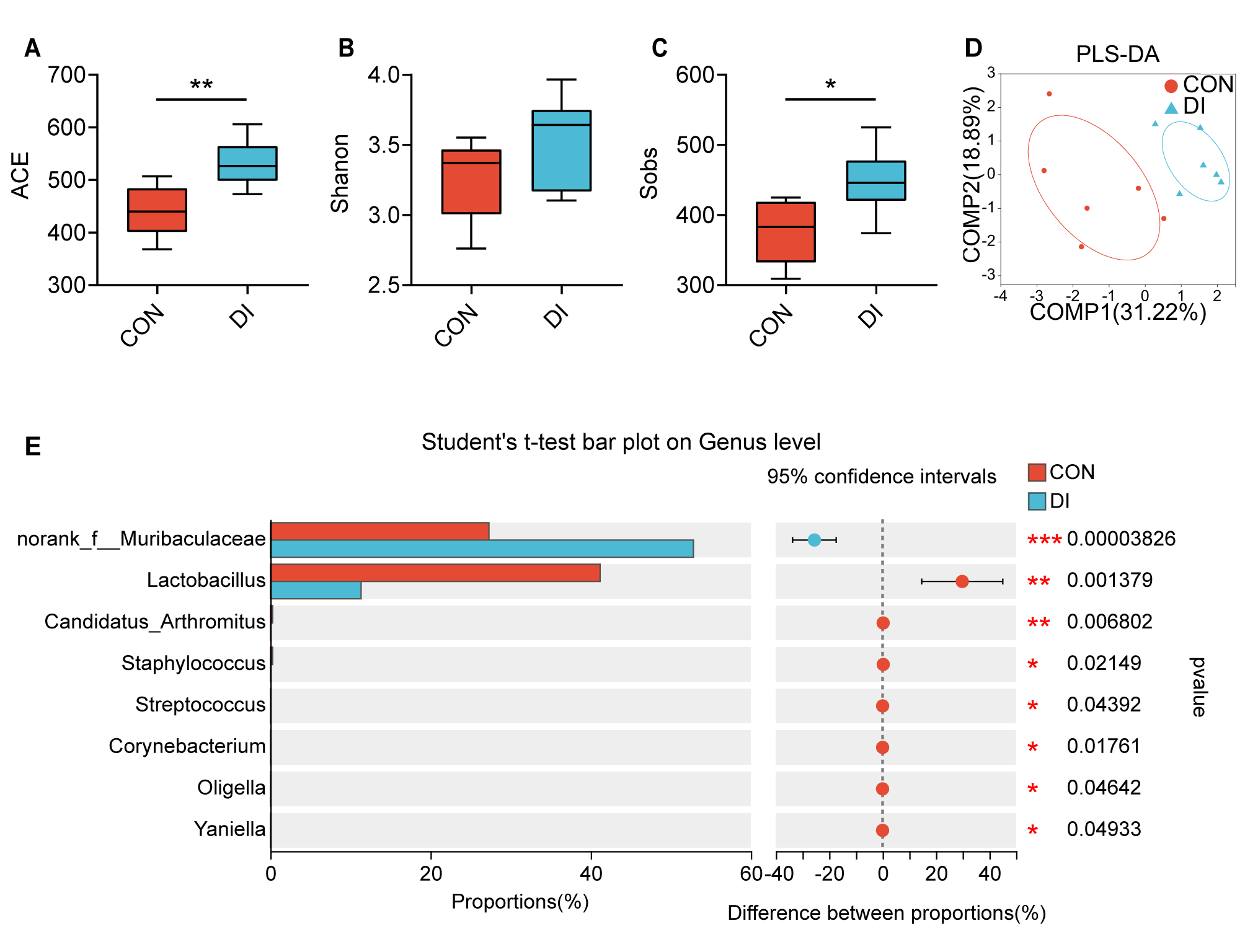


**Supplemental Figure 3.** Diversity among CON and DI groups. **(A-E)** Mice were given DI (400mg/kg) orally or distilled water for 7 days. Feces from the CON group and DI group mice were collected for 16S rRNA sequencing (n = 6). **(A-C)** Alpha diversity analysis of 16S rRNA sequencing data (ACE, Shanon and Sobs index). **(D)** Beta diversity analysis (PLS-DA). **(E)** Student's *t*-test bar plot. Data represent means ± SD; **P* < 0.05; ***P* < 0.01; ****P* < 0.001; by unpaired Student’s *t* test.

_­_**Supplemental Figure 4.** DI can lead to an enrichment of *Bacteroideta* and *norank_f_Muribaculaceae* in the fecal sample. **(A-G)** 0.5mM DI was thoroughly mixed with 50ml of MPYG culture medium, 1g of mouse feces was added, and the mixture was anaerobically incubated for 3 days. The precipitate was then subjected to 16S rRNA sequencing. (n = 3). **(A)** Fecal incubation experimental design. **(B)** The α-diversity analysis of gut microbes reflected by Shannon and Chao1 indices. **(C)** Scatter plots of weighted PCoA for the microbial composition showed the differences in gut microbial structure between the CON and DI groups. **(D)** Relative abundance of gut microbiota at the phylum level from different treatment groups. **(E)** Relative abundance of gut microbiota at the genus level from different treatment groups. **(F)** LEfSe showed different bacterial taxa that were enriched in different groups (log10 LDA score > 3.5). **(G)** The abundance of the genera *norank_f_Muribaculaceae* between the CON and DI groups. **P* < 0.05; ***P* < 0.01; ****P* < 0.001; compared with the CON group. Data represent means ± SD;


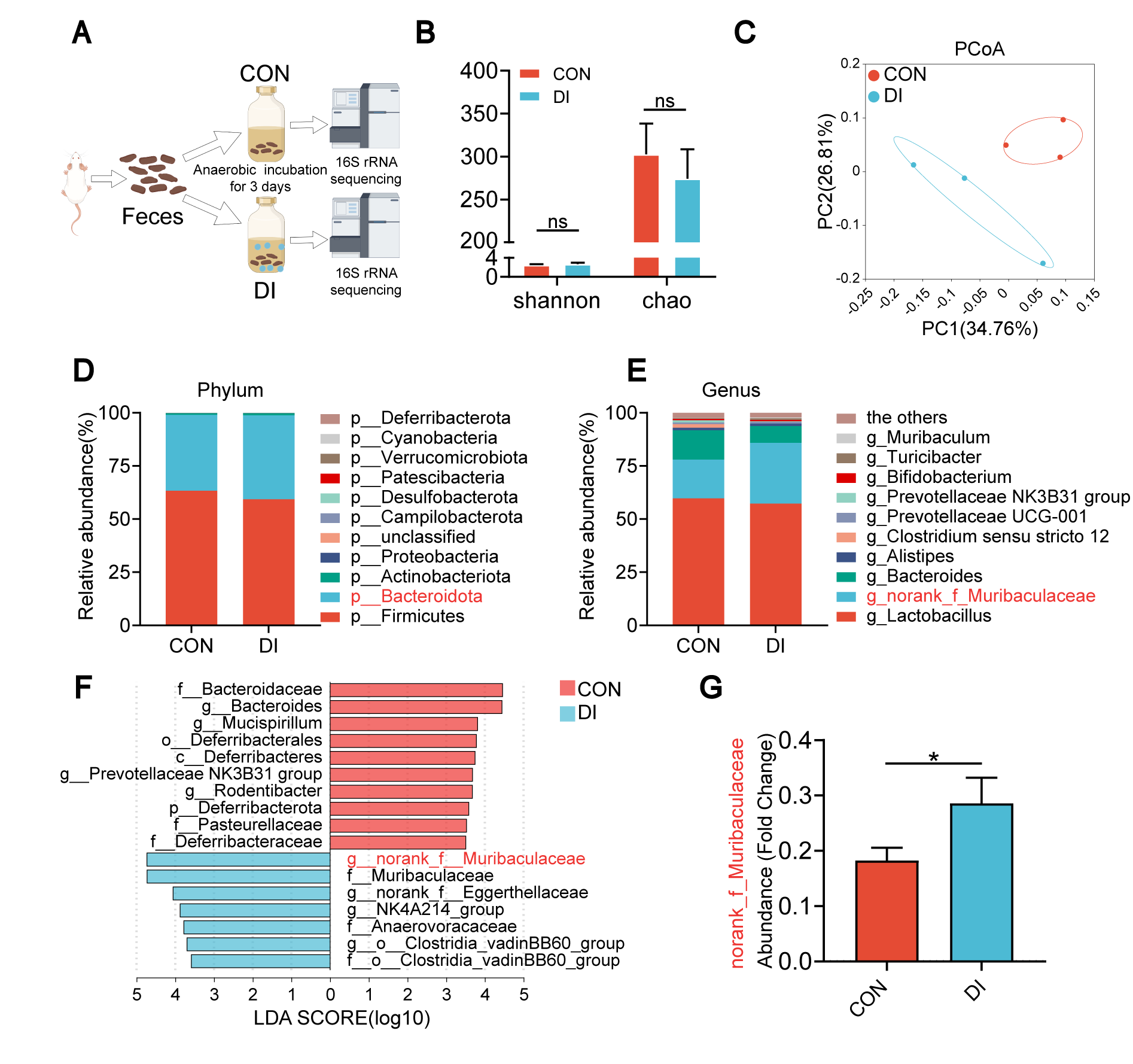

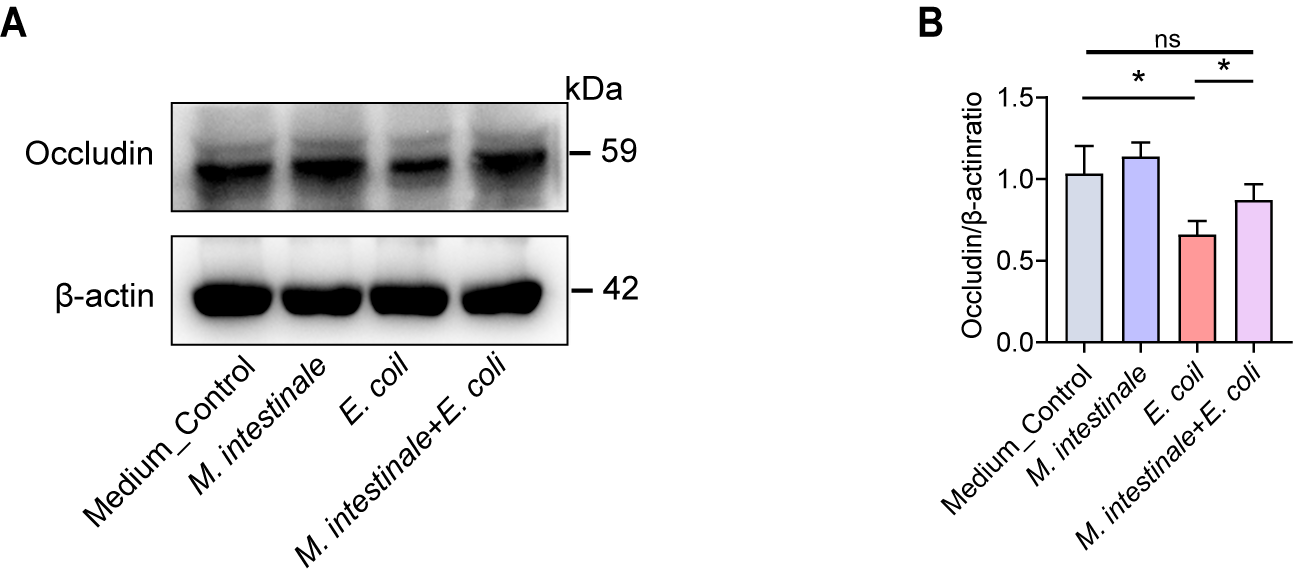


**Supplemental Figure5.** Changes in the levels of the tight junction protein Occludin in the *M. intestinale* administration experiment. **(A)** Representative Western blot images of Occludin in the uteri of mice and **(B)** analysis of the relative intensities (n = 3). Data represent means ± SD; **P* < 0.05; ***P* < 0.01; ****P* < 0.001; by unpaired Student’s *t* test.


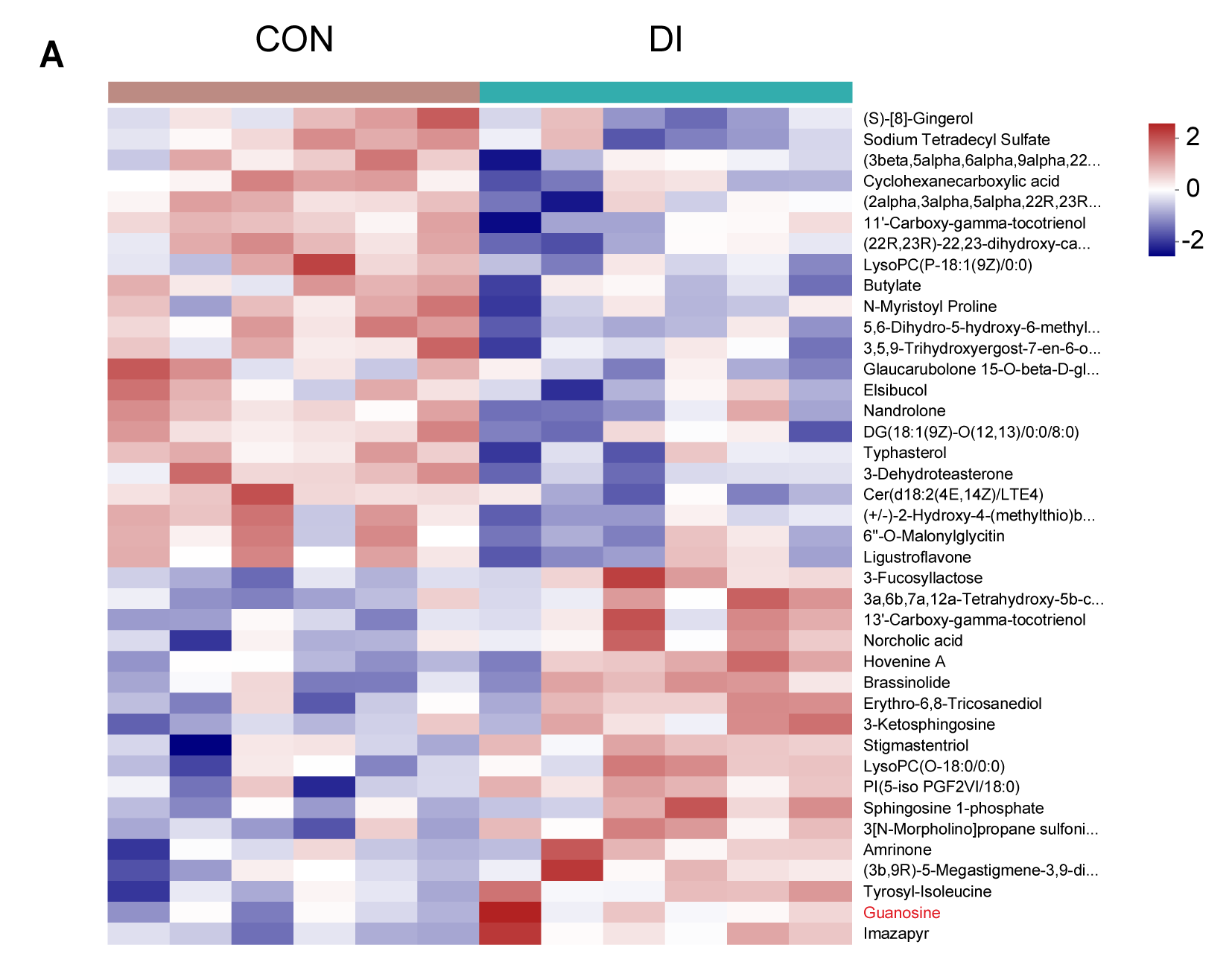


**Supplemental Figure 6.** Heat map of significantly altered metabolites (top40) in feces of CON group and DI group mice. **(A)** The relative content of guanosine in­­ the DI group was significantly higher than that in the CON group. n=6 individuals/group. Data were expressed as mean ± SD.


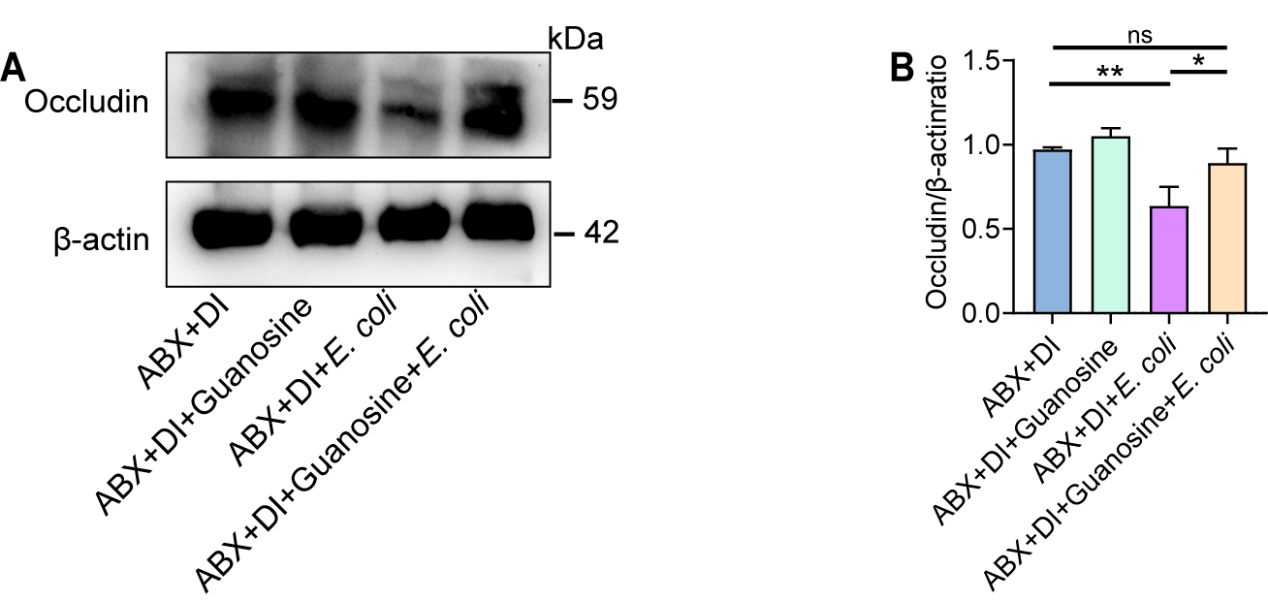


**Supplemental Figure 7.** Changes in the levels of the tight junction protein Occludin in the guanosine administration experiment. **(A)** Representative Western blot images of Occludin in the uteri of mice and **(B)** analysis of the relative intensities (n = 3). Data represent means ± SD; **P* < 0.05; ***P* < 0.01; ****P* < 0.001; by unpaired Student’s *t* test.


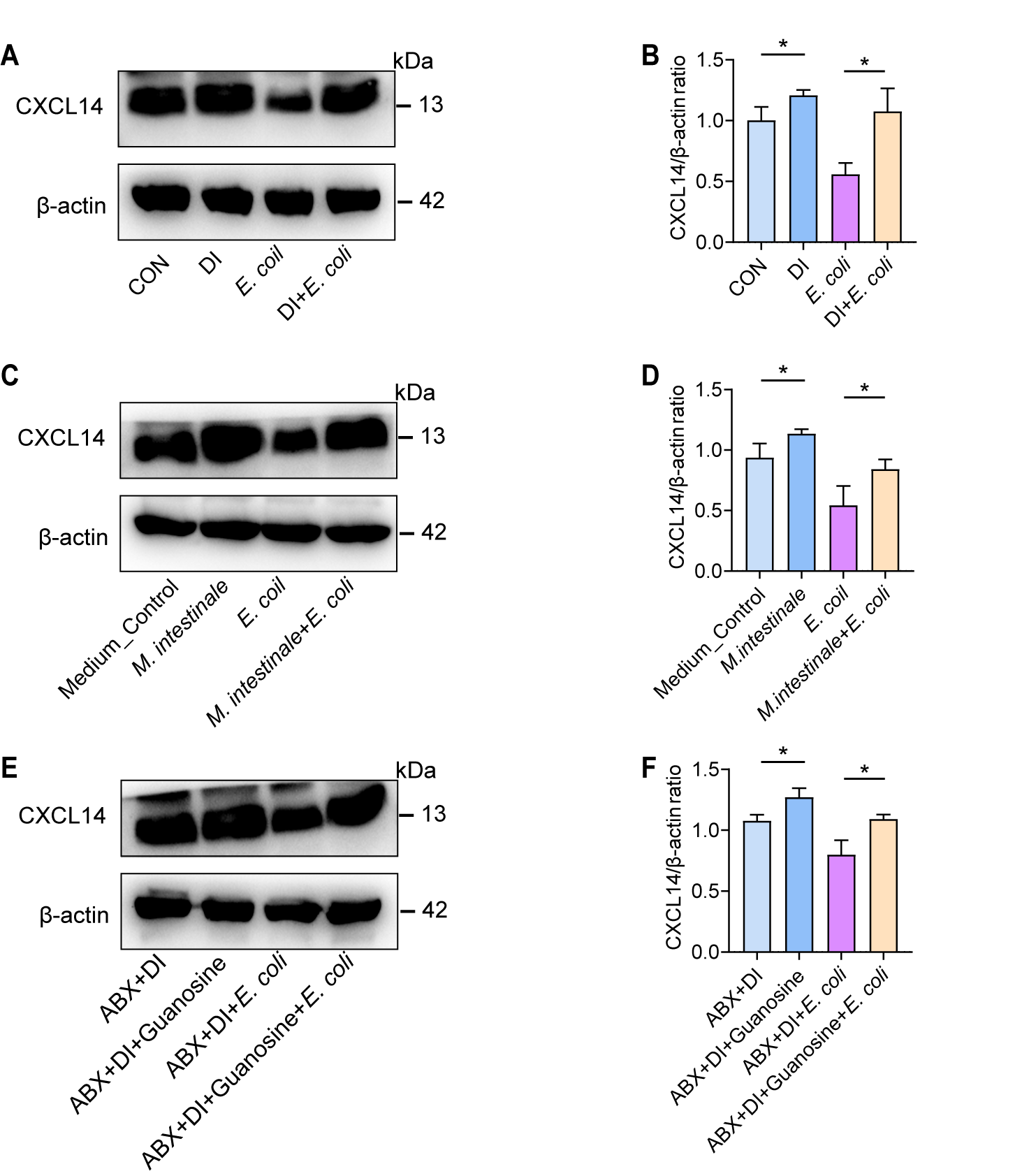


**Supplemental Figure 8.** DI significantly increases the protein level of CXCL14 in the uterus by regulating guanosine in a gut microbiota-dependent manner. **(A)** Representative Western blot images of CXCL14 in the uteri of mice in the DI administration experiment and **(B)** analysis of the relative intensities (n = 3). **(C)** Representative Western blot images of CXCL14 in the uteri of mice in the *M. intestinale* administration experiment and **(D)** analysis of the relative intensities (n = 3). **(E)** Representative Western blot images of CXCL14 in the uteri of mice in the guanosine administration experiment and **(F)** analysis of the relative intensities (n = 3). Data represent means ± SD; **P* < 0.05; ***P* < 0.01; ****P* < 0.001; by unpaired Student’s *t* test.


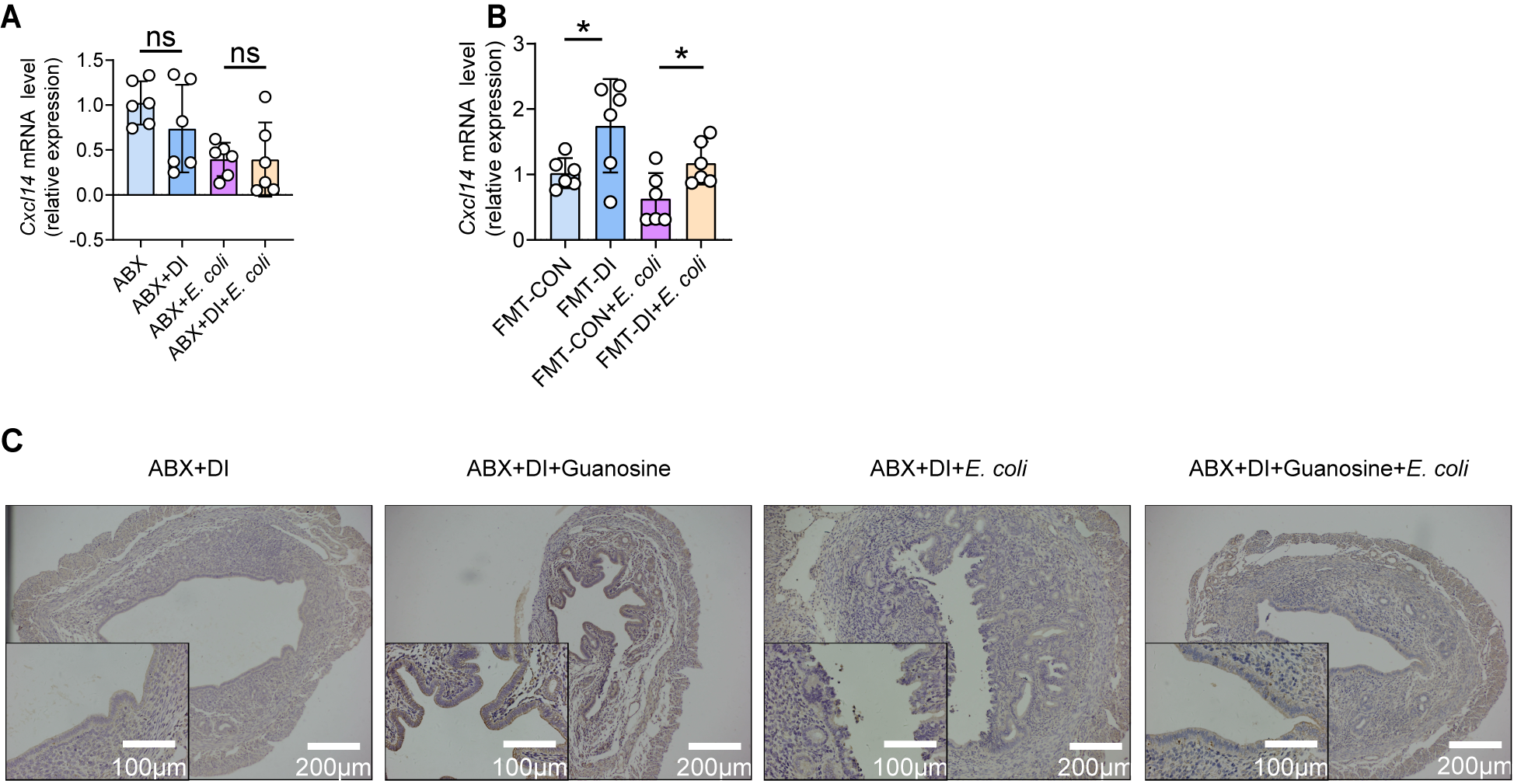


**Supplemental Figure 9.** DI significantly increases CXCL14 expression in uterine epithelial cells by regulating guanosine in a gut microbiota dependent manner. (**A-B)** Relative expression changes of CXCL14 in the ABX experiment and FMT experiment by Real-time PCR. **(C)** Representative images of immunohistochemistry staining with CXCL14. Data represent means ± SD; **P* < 0.05; ***P* < 0.01; ****P* < 0.001; by unpaired Student’s *t* test. The data shown are representative of three independent experiments


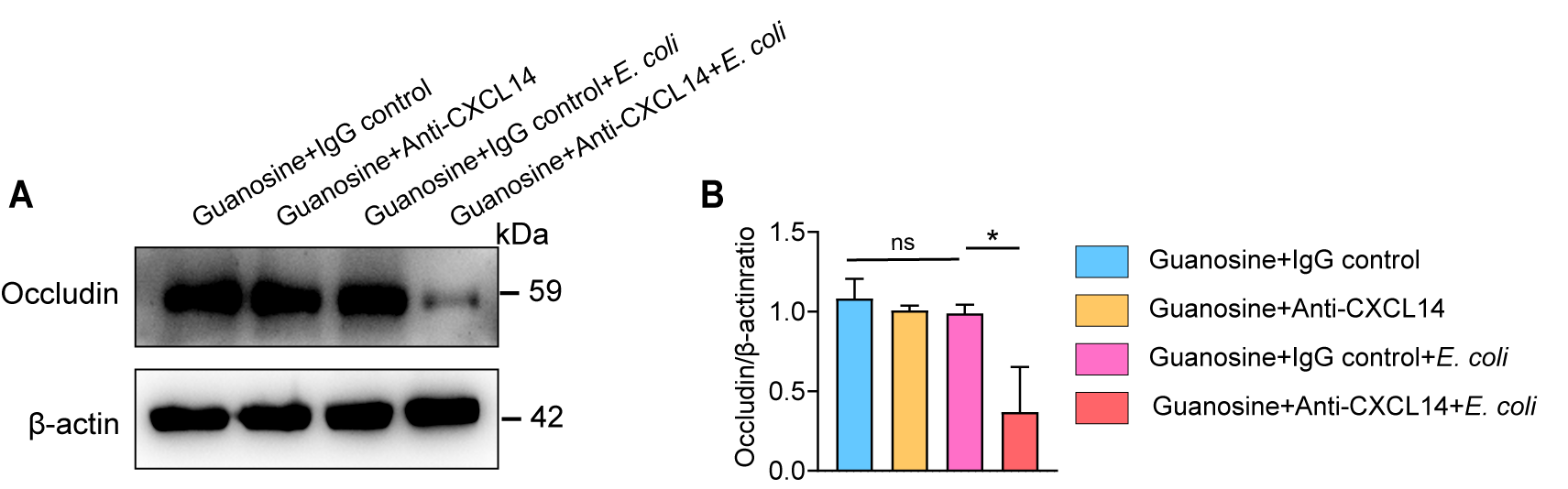


**Supplemental Figure 10.** Changes in the levels of the tight junction protein Occludin in the CXCL14 inhibition experiment. **(A)** Representative Western blot images of Occludin in the uteri of mice and **(B)** analysis of the relative intensities (n = 3). Data represent means ± SD; **P* < 0.05; ***P* < 0.01; ****P* < 0.001; by unpaired Student’s *t* test.

**Supplementary Table 1.** Primers information.

| **Gene** |  | **Sequences** |
| --- | --- | --- |
| GAPDH | Forward  Reverse | 5’-AACTTTGGCATTGTGGAAGG-3’  5′-ACACATTGGGGGTAGGAACA-3′ |
| IL-1β | Forward  Reverse | 5’-ACCTGTGTCTTTCCCGTGG-3’  5’-TCATCTCGGAGCCTGTAGTG-3’ |
| TNF-α | Forward  Reverse | 5’-TCTCATGCACCACCATCAAGGACT-3’  5’-ACCACTCTCCCTTTGCAGAACTCA-3’ |
| ZO-1 | Forward  Reverse | 5’-CAACATACAGTGACGCTTCACA-3’  5’-CACTATTGACGTTTCCCCACTC-3’ |
| Occludin | Forward  Reverse | 5’-CCCAGGCTTCTGGATCTATGT-3’  5’-TCCATCTTTCTTCGGGTTTTCA-3’ |
| CXCL14 | Forward  Reverse | 5’-TCCGGTCAGCATGAGGCTCC-3’  5’-CACCCTATTCTTCGTAGACC-3’ |
